# Climatology and dynamics of the link between dry intrusions and cold fronts during winter, Part II: Front-centred perspective

**DOI:** 10.1007/s00382-019-04793-2

**Published:** 2019-05-07

**Authors:** Shira Raveh-Rubin, Jennifer L. Catto

**Affiliations:** 1 Department of Earth and Planetary Sciences, Weizmann Institute of Science, Rehovot, Israel; 2 College of Engineering, Mathematics and Physical Sciences, University of Exeter, Exeter, UK

**Keywords:** Dry intrusions, Frontogenesis, Extratropical cyclones, Subtropical fronts, Composite analysis, Cold sector

## Abstract

The conceptual picture of an extratropical cyclone typically includes a cold front and a dry intrusion (DI) behind it. By objectively identifying fronts and DIs in ECMWF ERA-Interim data for 1979–2014, Part I quantified the climatological relationship between cold fronts and DIs. Driven by the finding that front intensity and frontal precipitation are enhanced in the presence of DIs, here we employ a front-centred perspective to focus on the dynamical and thermodynamical environment of cold fronts with and without DIs in the Northern Hemisphere winter. Distinguishing between trailing fronts (that connect to a parent cyclone) and isolated fronts, examples of DIs behind each type illustrate the baroclinic environment of the trailing front, and the lack of strong temperature gradients across the isolated front. Composite analyses of North Atlantic and North Pacific fronts outline the major differences in the presence of DIs, compared to similar fronts but without DIs in their vicinity. The magnitude and spatial structure of the modification by DIs depends on the front intensity. Yet, generally with DIs, trailing fronts occur with stronger SLP dipole, deeper upper-tropospheric trough, stronger 10-m wind gusts, enhanced ocean sensible and latent heat fluxes in the cyclone cold sector and heavier precipitation. Isolated weak fronts exhibit similar behaviour, with different spatial structure. This study highlights the central role of DIs for shaping the variability of fronts and their associated environment and impact.

**Electronic supplementary material:**

The online version of this article (10.1007/s00382-019-04793-2) contains supplementary material, which is available to authorized users.

## Introduction

For almost a century have cyclones and their attendant fronts been recognized as the main impact-producing weather system in the extratropics (Bjerknes and Solberg 1922). Numerous observational and numerical case studies have examined the passage of cold fronts trailing from midlatitude cyclones, and their link to precipitation, convection and mesoscale systems of high impact (e.g., Browning et al. 1973; Hobbs et al. 1980; Doswell 1987; Neiman and Shapiro 1993; Browning and Roberts 1994a). Conceptual models help to summarize the 3D geometry of cyclones, fronts and associated airstreams, their evolution and dynamics that generate high-impact weather across the the synoptic- and mesoscale (Carlson 1980; Browning 1986; Shapiro and Keyser 1989; Browning 1997).

Composites are an invaluable tool for revealing mean characteristics of the front or cyclone environment (e.g. Catto et al. 2010), suggesting coherent climatological-statistical patterns. For example, by combining composite satellite and reanalysis data for cyclones of different strengths, Field and Wood (2007) show that intense cyclones are associated with increased precipitation compared to less intense cyclones. The cold sector of cyclones, i.e., the post-frontal region is characterized by low clouds, high wind speeds (Field and Wood 2007; Naud et al. 2016) and negative potential vorticity due to reduced static stability in the boundary layer (Vanniere et al. 2016).

Recently, global climatological quantification of front occurrence has been carried out based on objective front identification in reanalysis data (Berry et al. 2011; Catto et al. 2012; Simmonds et al. 2012; Papritz et al. 2014; Rudeva and Simmonds 2015; Schemm et al. 2015). Climatologically relating fronts to precipitation indicates that the majority of oceanic storm track precipitation is associated with cold fronts (Catto et al. 2012). Moreover, most midlatitude extreme precipitation events are associated with fronts, while mean front intensities, in terms of thermal gradients across the front, are stronger during extreme precipitation, compared to other precipitation-producing fronts (Catto and Pfahl 2013).

In the cold sector of cyclones, dry intrusion (DI) airstreams descend equatorward and eastwards towards the cold front (Browning 1997; Wernli 1997). Dry intrusions are also an inegral part of the extratropical transition (ET) warm seclusion life cycle of cyclones (Dekker et al. 2018), and are associated with frontogenesis during the re-intensification phase of an ET of a typhoon (Zhu et al. 2018). DIs may transport ozone to the middle and lower troposphere (Jaeglé et al. 2017), and are generally associated with enhanced surface winds (Kolstad 2015; Raveh-Rubin and Wernli 2015, 2016; Hart et al. 2017). The DI air often mixes with the underlying boundary layer, affecting strongly the moisture and temperature distributions and potentially sharpening the temperature and moisture gradients across the front. Thus, DIs potentially sharpen temperature and humidity gradients across low-level fronts and increase their intensity. In response to the incoming dry and cold air, surface fluxes can be triggered (Raveh-Rubin 2017; Aemisegger and Papritz 2018). Convective activity may be enhanced by overrunning of the dry airmass and inducing potential instability. Therefore, the response of precipitation to the existence of DIs may be a combination of reduced precipitation due to low-level drying and evaporation of raindrops falling from the cloud head into the dry airmass, but at the same time, convective precipitation may be favoured (Browning and Reynolds 1994; Browning and Roberts 1994b, 1996; Bethan et al. 1998; Gao et al. 2010). To date, the climatological association between cold fronts and DIs has not been carried out, impeding our understanding of the net effect of DIs on fronts and their cold sector as well as their associated wind and precipitation impact.

In the subtropics fronts have gained considerably less attention, although they compose a primary element of high-impact weather (Catto and Pfahl 2013; Dowdy and Catto 2017) and influence the surface energy balance (Beringer and Tapper 2000) and convective activity (Reeder and Smith 1998). Fronts are identified in the subtropics, away from the storm track regions, in the Eastern North Pacific, Southern Indian Ocean, north of Australia and in South America (Berry et al. 2011). Case studies suggest that in some cases subtropical fronts are directly related to upper-level midlatitude cutoff low and a tropopause fold (Griffiths et al. 1998), midlatitude low (Deslandes et al. 1999) or to weak baroclinicity and a shallow density current at the edge of a system at higher latitude (Trier et al. 1990). However, in other cases, the front may not be related to a midlatitude baroclinic environment (Reeder et al. 2000).

The first climatological quantification of the co-occurrence of DIs and cold fronts is provided in Part I of this paper (Catto and Raveh-Rubin 2019). Statistically stronger front intensities in the presence of DIs, as well as longer fronts and enhanced precipitation are found both for fronts trailing from cyclones as well as for isolated fronts, that do not connect to any cyclone area of influence. Process understanding of the dynamical interaction between DIs and fronts is needed to further interpret the statistical relationships, with the aid of investigating the dynamical environment near the fronts. The emerging questions we aim to address here are: What is the dynamical environment of DIs matching with cold fronts? Are there common patterns for trailing and isolated fronts even though they are fundamentally different in terms of their dynamical environment? What is the climatological dynamical environment of trailing and isolated fronts that occur together with DIs, and how it is different from the environment of similar fronts that occur without DIs? Are fronts occurring together with DIs more impactful (in terms of precipitation and wind gusts)?

Here, we will address these questions by investigating trailing and isolated fronts separately. For each, a case study will exemplify the three-dimensional environment of the matching DI and cold front. Further, a composite of a large number of fronts will highlight general environmental characteristics of the interaction for the Northern Hemisphere winter. The objective identification algorithm and composite methodology are described in Sect. 2; Results of DI occurrence with trailing and isolated fronts are presented in Sects. 3 and 4, respectively. The results are then summarized and further discussed in Sect. 5.

## Data and methodology

Analysis of atmospheric fields is based on the ERA-Interim reanalysis of the European Centre for Medium-range Weather Forecasts (Dee et al. 2011), available with 6-hourly time intervals, 60 hybrid vertical levels and interpolated to a regular $1^\circ \times {} 1^\circ$ horizontal grid, for the months December, January, February (DJF) for the years 1979–2014. Surface fluxes and precipitation fields are accumulated over the preceding 6 h, and the 10-m wind gust represents the maximum value over the preceding 3 h.

*Feature-based identification algorithms*

Dry intrusions are identified in a two-step procedure. First, Lagrangian-based air-parcel trajectories are computed according to the ERA-Interim wind field, using the Lagrangian analysis tool LAGRANTO version 2.0 (Sprenger and Wernli 2015). The construction of the Lagrangian DI dataset is composed of a systematic calculation of forward trajectories from a regular global grid with 80 km intervals in the horizontal and 20 hPa in the vertical (above 600 hPa) every 6 h, followed by a selection of DI trajectories as the ones that descend at least 400 hPa within any 48-h period (Raveh-Rubin 2017). In the second step, the DI trajectories are regridded into a $1^{\circ } \times {} 1^{\circ }$ grid using a nearest neighbour method, whenever their pressure is equal to or higher than 700 hPa. The resulting two-dimensional low-level Eulerian DI objects are converted into a binary mask, i.e., a grid box is considered to be occupied by a low-level DI if the number of DI trajectories exceeds $10^{-5}$ trajectories per $\hbox {km}^{2}$.

Fronts are identified according to the thermal front parameter based on the gradients of wet-bulb potential temperature field at 850 hPa, following Hewson (1998) and Berry et al. (2011). The front type (warm or cold) is then identified according to its thermal advection. The grid points with identified fronts are then connected to form line objects using a search radius of $3^{\circ }$, and further expanded by two grid points in all directions to allow a matching with the low-level DI objects typically found adjacent to them. Two particular types of cold fronts will be the focus of the current analysis: cold fronts trailing from extratropical cyclones, and isolated cold fronts that cannot be associated with a cyclone. These types of cold fronts will be termed for simplicity ‘trailing fronts’ and ‘isolated fronts’ throughout the manuscript. The attribution of a front type uses automatically-identified cyclone masks, taken as the outermost closed contour around sea-level pressure minima, using 0.5-hPa contour intervals, adapted from Wernli and Schwierz (2006). Trailing fronts are spatially connected with a cyclone area, but are defined outside of this central area of the cyclone, while isolated fronts are not connected at all to any cyclone mask (e.g., Figs. 2, 8 and the supplementary animation). The reader is encouraged to refer to Part I of this work (Catto and Raveh-Rubin 2019) for additional details on the identification and matching algorithm, its sensitivity to its controlling parameters and the resulting climatologies.

**Fig. 1:**
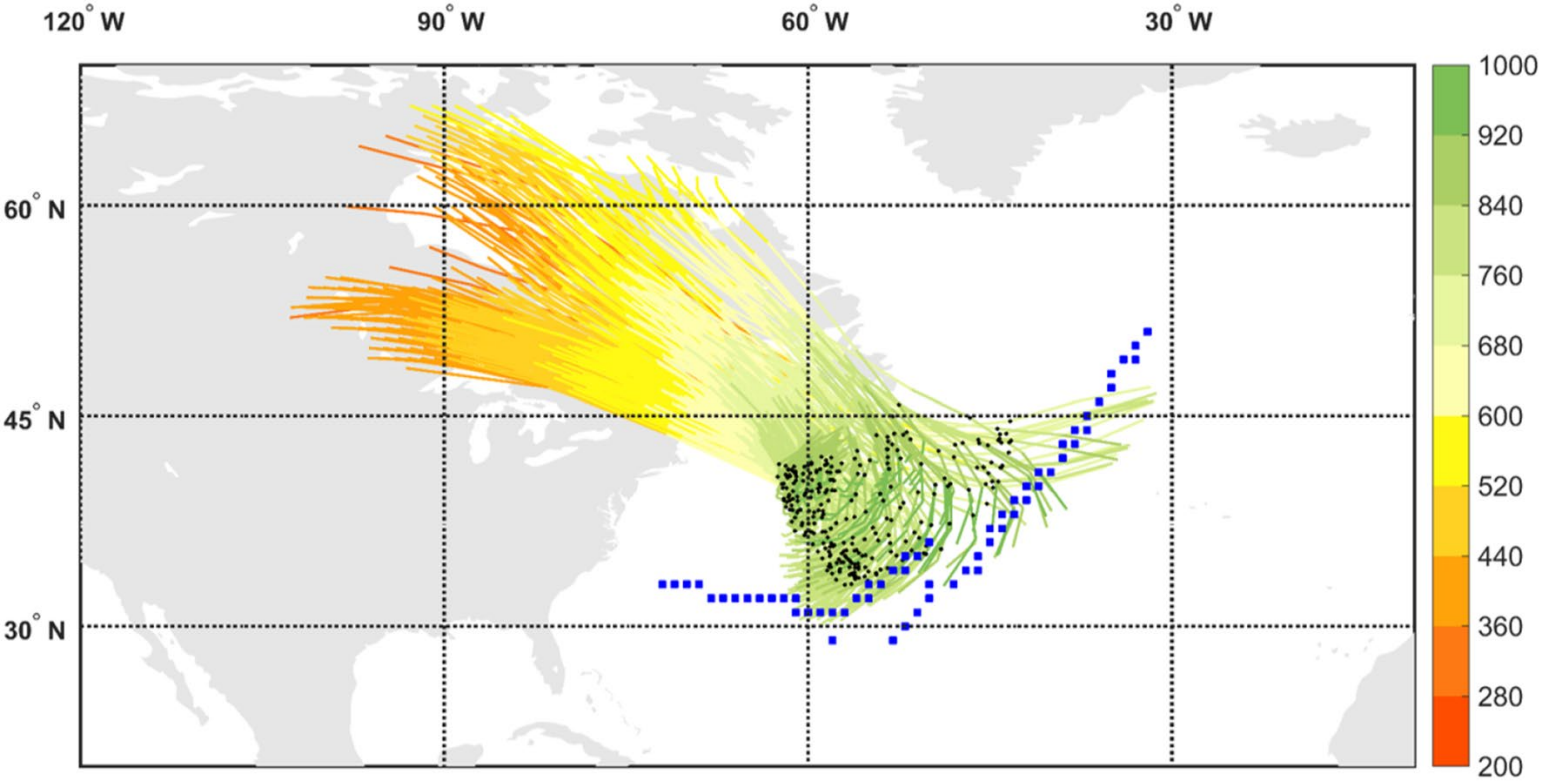
Dry intrusion trajectories, coloured according to their pressure (hPa), and the cold trailing front it is associated with at 00 UTC 3 January 2005 (blue squares). The DI trajectories start their 400-hPa descent at 12 UTC 1 January 2005, and end their descent at 12 UTC 3 January 2005. The black dots mark the location along the trajectories at the timing that corresponds to the marked front (i.e., 36 h after the DI start)

**Fig. 2:**
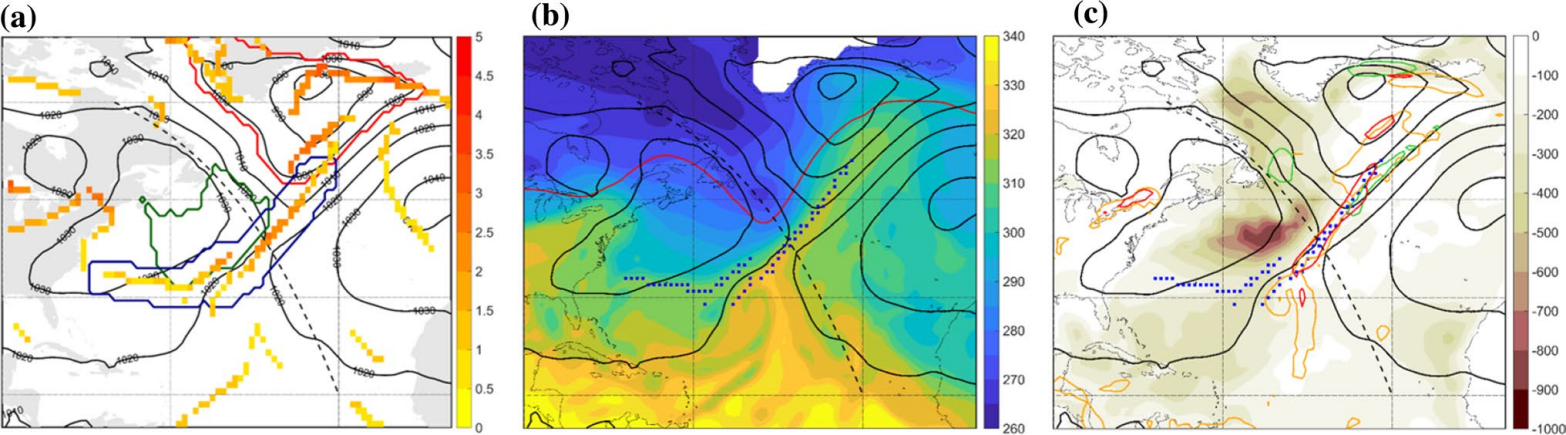
Trailing front case at 00 UTC 3 January 2005 showing sea-level pressure (hPa, black) and **a** all identified front grid points coloured according to their intensity (K/100 km). The red contour marks the cyclone area, the blue contour marks the expanded trailing front, the green contours marks the DI object matched with this front, **b** equivalent potential temperature on 850 hPa (K, shaded), isentropic PV on the 320-K surface (2-PVU in red contour line), **c** sea-surface heat fluxes into the atmosphere (sensible + latent, $\hbox {W m}^{-2}$), 3-h 10-m wind gusts maximum ($25\,\hbox { m s}^{-1}$, green contour), 6-h accumulated convective precipitation (1.6 mm, orange contour) and 6-h accumulated total precipitation (8 mm, red contour). The trailing front matching the DI is marked with blue squares in **b** and **c**, and the dashed line in all panels marks the location of the vertical cross section in Fig. 3

**Fig. 3:**
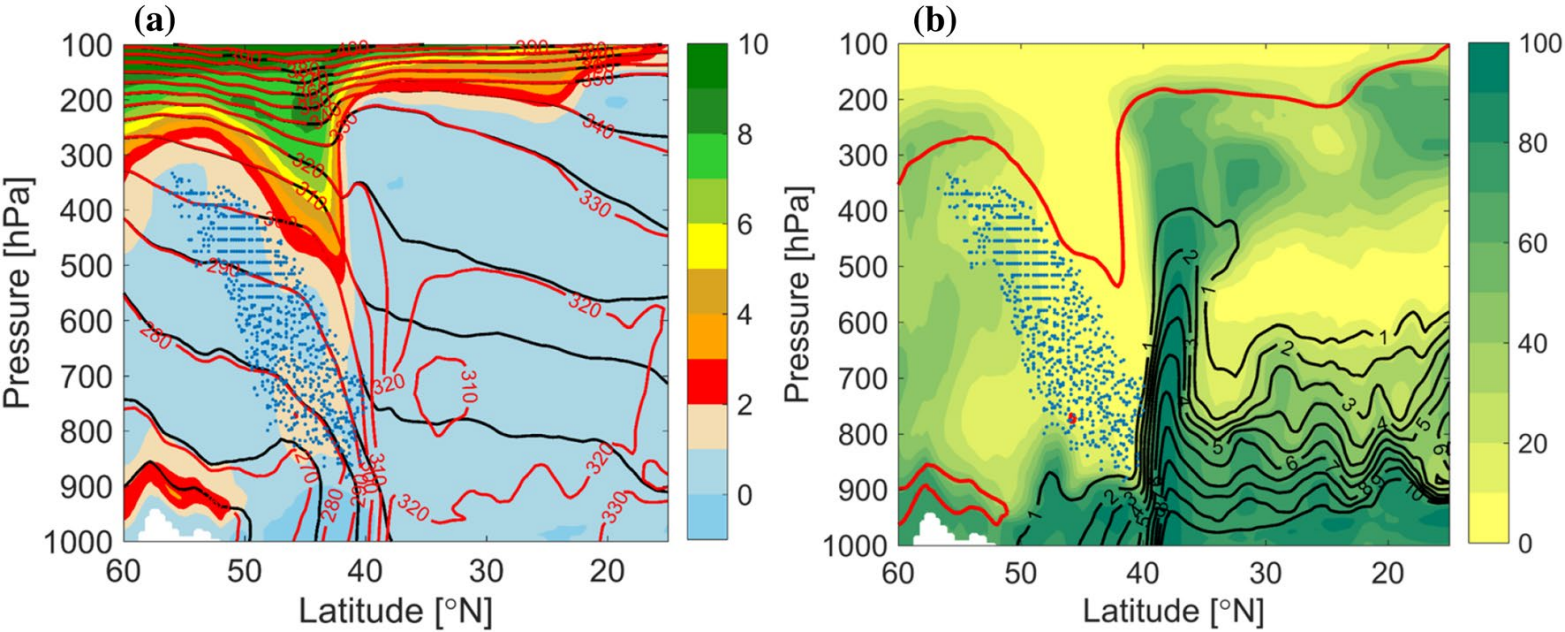
Vertical cross sections along the dashed line shown in Fig. 2. **a** PV (PVU, shaded), potential temperature (black contour) and equivalent potential temperature (K, red contour). **b** Relative humidity (%, shaded), specific humidity ($\hbox {g kg}^{-1}$, black contour) and 2-PVU contour (red). The blue points in both panels mark the intersection points of the DI trajectories with the vertical cross section, i.e., at any phase during their 48-h time of descent, and starting at different times

**Fig. 4:**
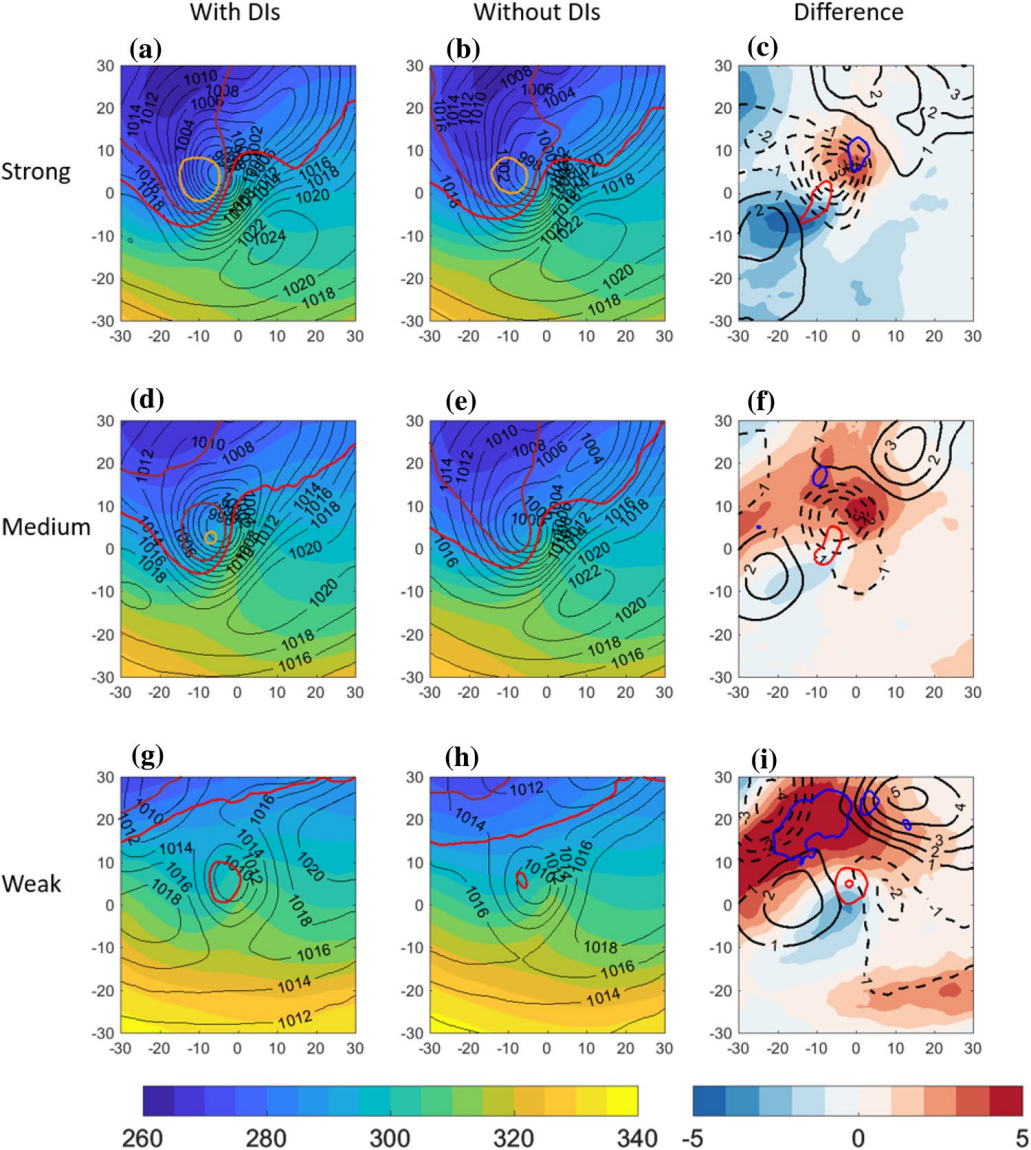
Centered composite of North Atlantic cold trailing fronts, centered around their NE corner at [$0^{\circ }\hbox {E},0^{\circ }\hbox {N}$] relative longitude and latitude, for different front sets. Top row: strong front intensity; middle row: medium front intensity; bottom row: weak front intensity. Left column: composite of cold trailing fronts that match with a DI; middle column: cold trailing fronts that do *not* match with a DI. Right column: difference between composite means [with DIs]–[without DIs]. Plotted for the full fields are $\theta _{e}$ on 850 hPa (K, shaded), sea-level pressure (hPa, black contour), and potential vorticity on 300 hPa (2 PVU in red, 3 PVU in brown and 4 PVU in orange). Plotted for the difference fields are $\theta _{e}$ on 850 hPa (K, shaded), SLP (hPa, solid and dashed black lines, zero line omitted), and PV on 300 hPa (PVU, red contours for positive values above 0.5 PVU at 0.5 PVU intervals, and blue contours for the negative counterpart, 0-contour omitted)

**Fig. 5:**
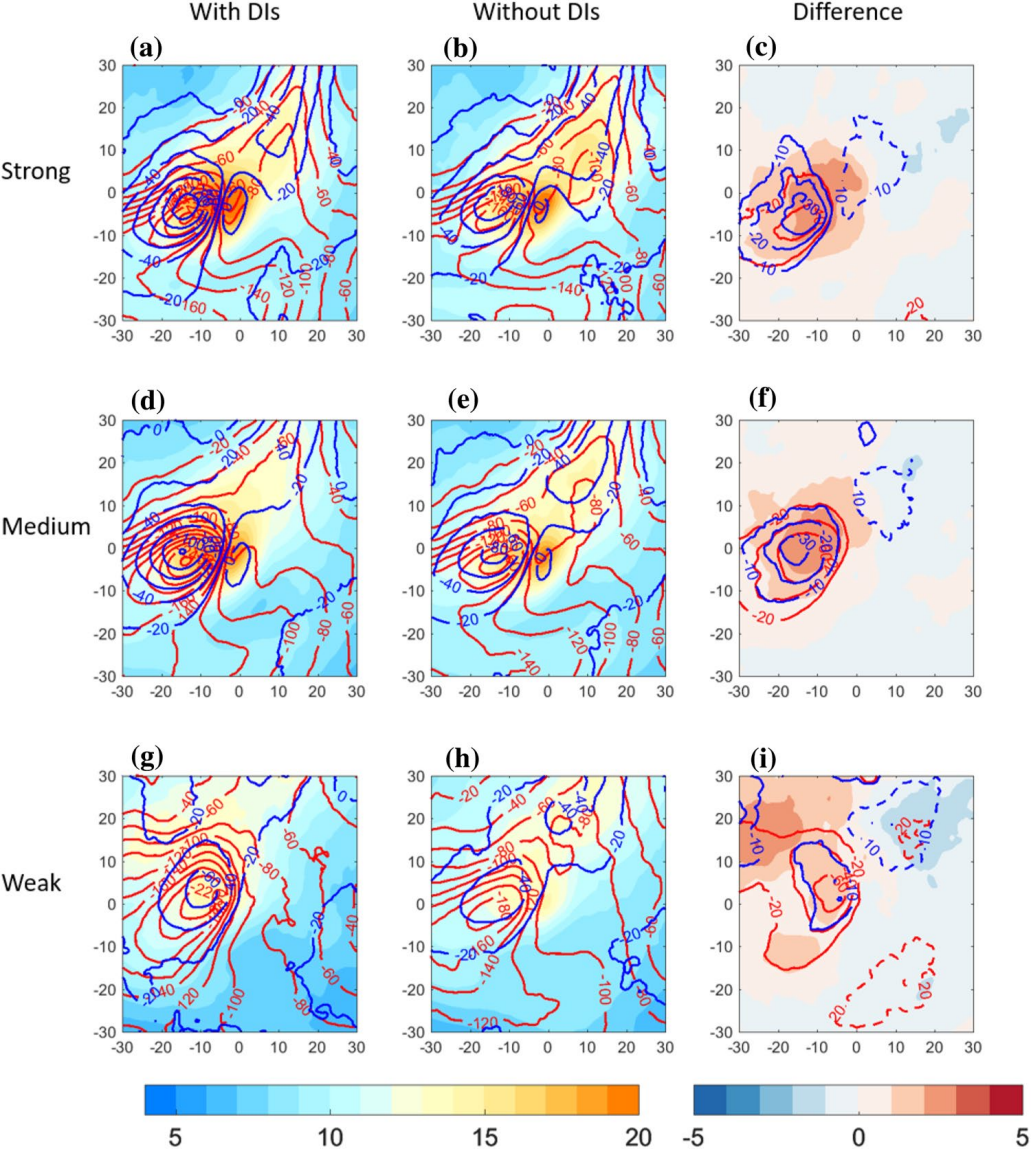
Centered composite of North Atlantic cold trailing fronts, centered around their NE corner at [$0^{\circ }\hbox {E}, 0^{\circ }\hbox {N}$] relative longitude and latitude, for different front sets. Top row: strong front intensity; middle row: medium front intensity; bottom row: weak front intensity. Left column: composite of cold trailing fronts that match with a DI; middle column: cold trailing fronts that do *not* match with a DI. Right column: difference between composite means [with DIs]–[without DIs]. Plotted for the full fields are maximum 10-m wind gust ($\hbox {m s}^{-1}$, shaded), surface sensible heat flux ($\hbox {W m}^{-2}$, blue) and surface latent heat flux ($\hbox {W m}^{-2}$, red). Plotted for the difference fields are maximum 10-m wind gust ($\hbox {m s}^{-1}$, shaded), surface sensible heat flux ($\hbox {W m}^{-2}$, blue contour with $10\hbox { W m}^{-2}$ interval, solid line for negative and dashed line for positive values) and surface latent heat flux ($\hbox {W m}^{-2}$, red contour with $20\hbox { W m}^{-2}$ interval, solid line for negative and dashed line for positive values)

**Fig. 6:**
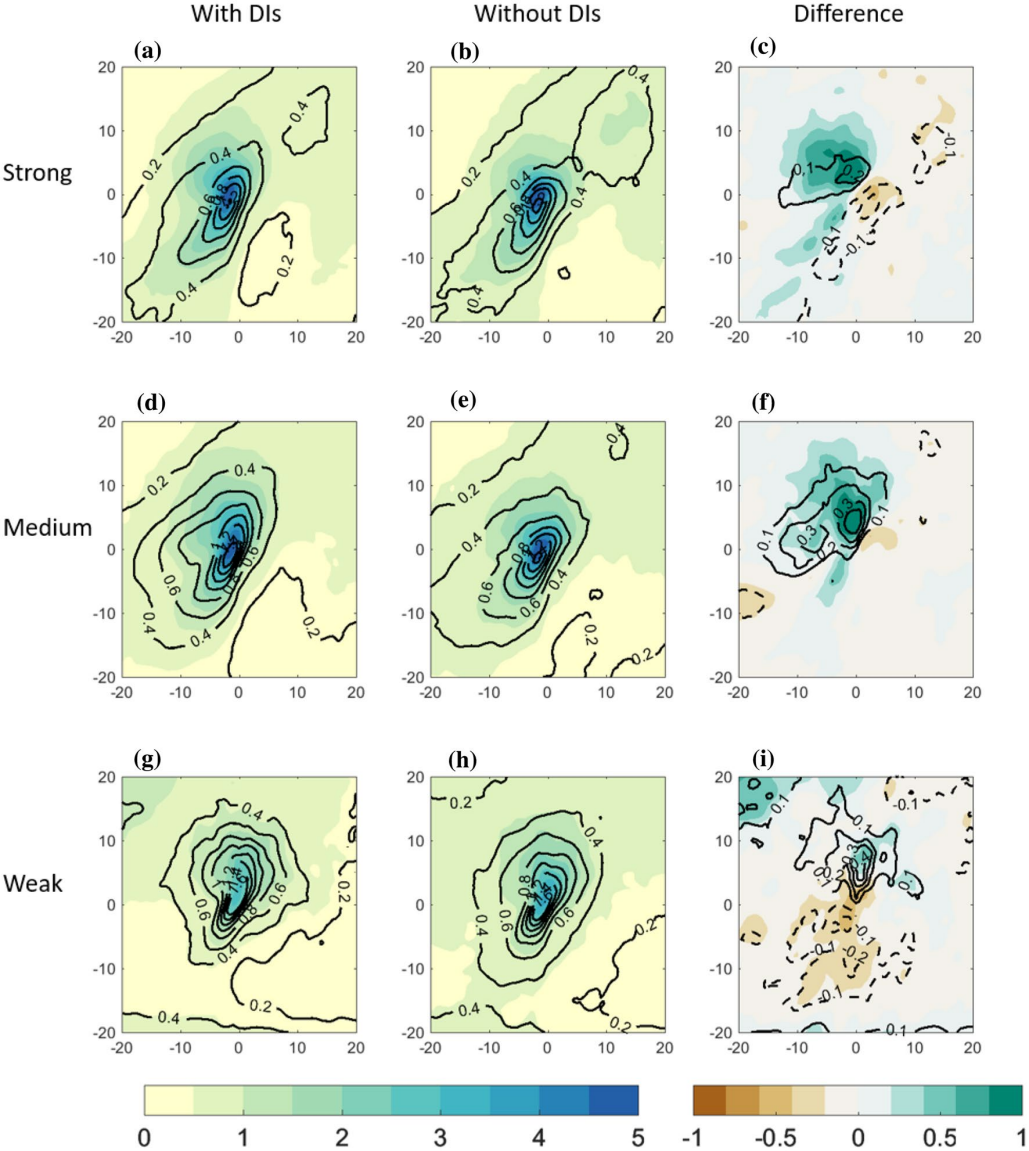
Centered composite of North Atlantic cold trailing fronts, centered around their NE corner at [$0^{\circ }\hbox {E}, 0^{\circ }\hbox {N}$] relative longitude and latitude, for different front sets. Top row: strong front intensity; middle row: medium front intensity; bottom row: weak front intensity. Left column: composite of cold trailing fronts that match with a DI; middle column: cold trailing fronts that do *not* match with a DI. Right column: difference between composite means [with DIs]–[without DIs]. Plotted are total precipitation ($\hbox {mm (6h)}^{-1}$, shaded) and convective precipitation ($\hbox {mm (6h)}^{-1}$ in black). Note that the spatial extent covers $\pm \, 20^{\circ }$ , in contrast to $\pm \,30^{\circ }$ in Figs. 4, 5)

**Fig. 7:**
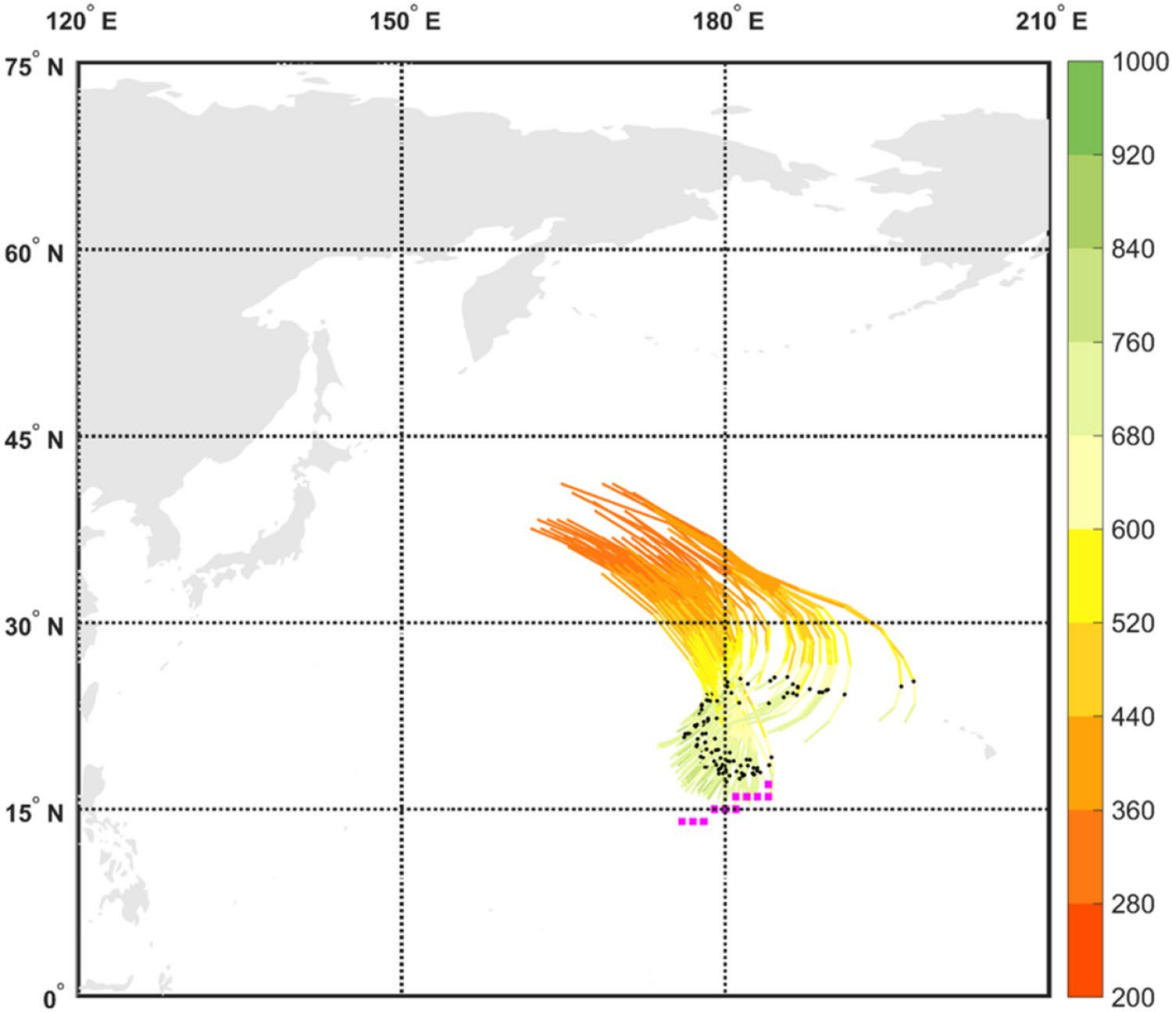
Dry intrusion trajectories, coloured according to their pressure (hPa), and the cold isolated front it is associated with at 00 UTC 12 February 2005 (pink squares). The DI trajectories start their 400-hPa descent at 12 UTC 10 February 2005, and end their descent at 12 UTC 12 February 2005. The black dots mark the location along the trajectories at the timing that corresponds to the marked front (i.e., 36 h after the DI start)

*Compositing methodology*

In order to learn about coherent differences between the dynamical and thermodynamical environment of different fronts, compositing is applied for subsets of trailing and isolated front objects occurring in December, January and February (DJF) between 1979 and 2014. Compositing is performed separately for the North Pacific and North Atlantic regions, i.e., for the central ocean basins in the Northern Hemisphere to avoid the influence of land masses. Front objects are considered for the composites if they have at least one grid point within the North Pacific rectangle 20–40$^{\circ }\hbox {N}$ and 150–210$^{\circ }\hbox {E}$ or North Atlantic rectangle 20–40$^{\circ }\hbox {N}$ and 60–30$^{\circ }\hbox {W}$. Several compositing methodologies have been used in the past to examine structures of interest, by applying rotation, transformation and/or rescaling of extratropical cyclones (e.g., Field and Wood 2007; Rudeva and Gulev 2011) or fronts (Naud et al. 2016). Here, we consider a large number of fronts, aiming to study their large-scale environment and potential differences of this environment among the different front sets, varying in location, front type, front strength, and the presence of DIs. Therefore, to maintain a focus on the fronts and examine the front-relative environment, the composites are centered around the northeastern corner of the expanded front objects, and extend $\pm \, 30^{\circ }$ of longitude and latitude. The front angle, measured as the azimuth of the line connecting the two front end points, varies by 20–25$^{\circ }$ (for trailing fronts) and 20–30$^{\circ }$ (for isolated fronts). Since we are interested in the front environment, rather than the front structure itself, we do not further introduce a rotation of the composited field according to the front angle. Introducing a rotation factor into the compositing procedure enhances the front composite sharpness, but at the same time adds uncertainties to the composite patterns away from the front, and obscures the realistic orientation of the meridional baroclinic structure and the associated surface pressure patterns. Yet, the front structure is clearly visible in the composites of 850-hPa $\theta _{e}$ (Figs. 4, 10). Importantly, front intensity is significantly higher when it is accompanied by a DI (Catto and Raveh-Rubin 2019). Therefore, to study the specific aspects that relate to DIs, the front intensity is controlled by separating the composite analysis to three distinct front intensities (measured as the maximal 850-hPa $\theta _{w}$ gradient across the front), namely strong (2.5–3.0 K/100 km), medium (1.8–2.2 K/100 km) and weak (1.0–1.5 K/100 km). Separate composites are then examined for trailing and isolated fronts, with and without matching DIs. Tables 1 and 2 summarize the resulting number of front objects in each category. Please note that the intensity measure is based on wet-bulb potential temperature field, which varies less than equivalent potential temperature, therefore resulting in apparently smaller gradients compared to fronts identified using equivalent potential temperature (e.g., Schemm et al. 2015; Spensberger and Sprenger 2018).

**Fig. 8:**
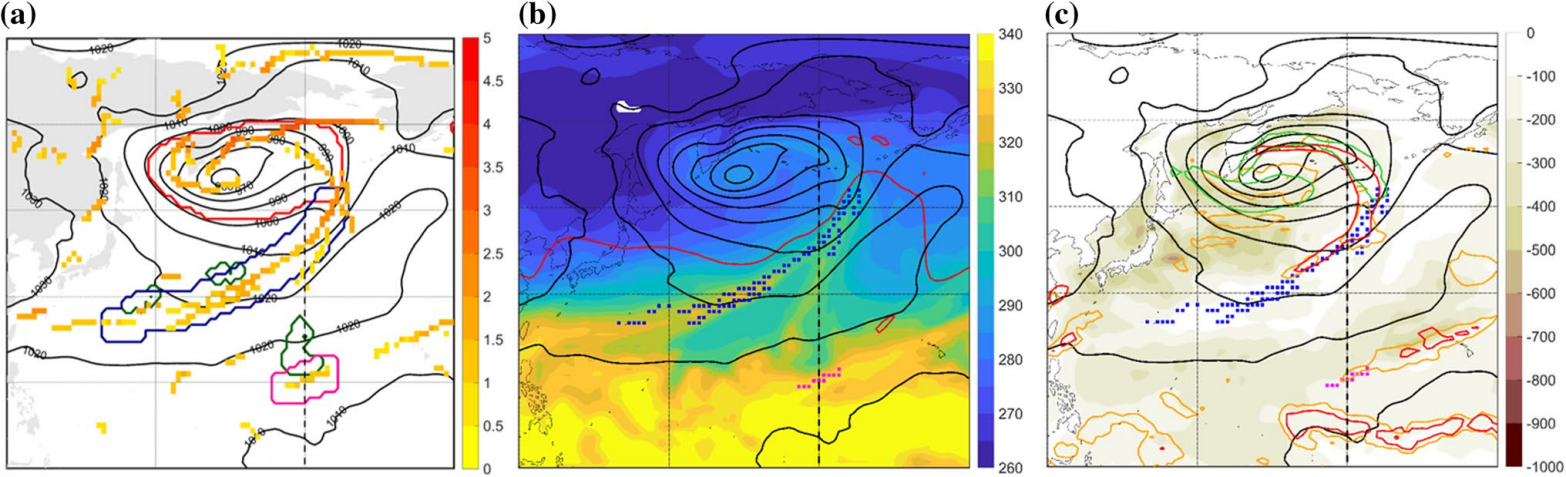
Isolated front case at 00 UTC 12 February 2005 showing sea-level pressure (hPa, black) and **a** all identified front grid points coloured according to their intensity (K/100 km). The red contour marks the cyclone area, the pink contour marks the expanded isolated front, the green contours marks the DI object matched with this front. Additionally, a nearby trailing front (blue contour) matches with two DI objects (green contour), **b** equivalent potential temperature on 850 hPa (K, shaded), isentropic PV on the 320-K surface (2-PVU in red contour line), **c** sea-surface heat fluxes into the atmosphere (sensible + latent, $\hbox {W m}^{-2}$), 3-h 10-m wind gusts maximum ($25\hbox { m s}^{-1}$, green contour), 6-h accumulated convective precipitation (1.6 mm, orange contour) and 6-h accumulated total precipitation (8 mm, red contour). The isolated front in focus is marked with the pink squares and the nearby trailing front is marked with blue squares in **b** and **c**, the dashed line along the dateline in all panels marks the location of the vertical cross section in Fig. 9

**Fig. 9:**
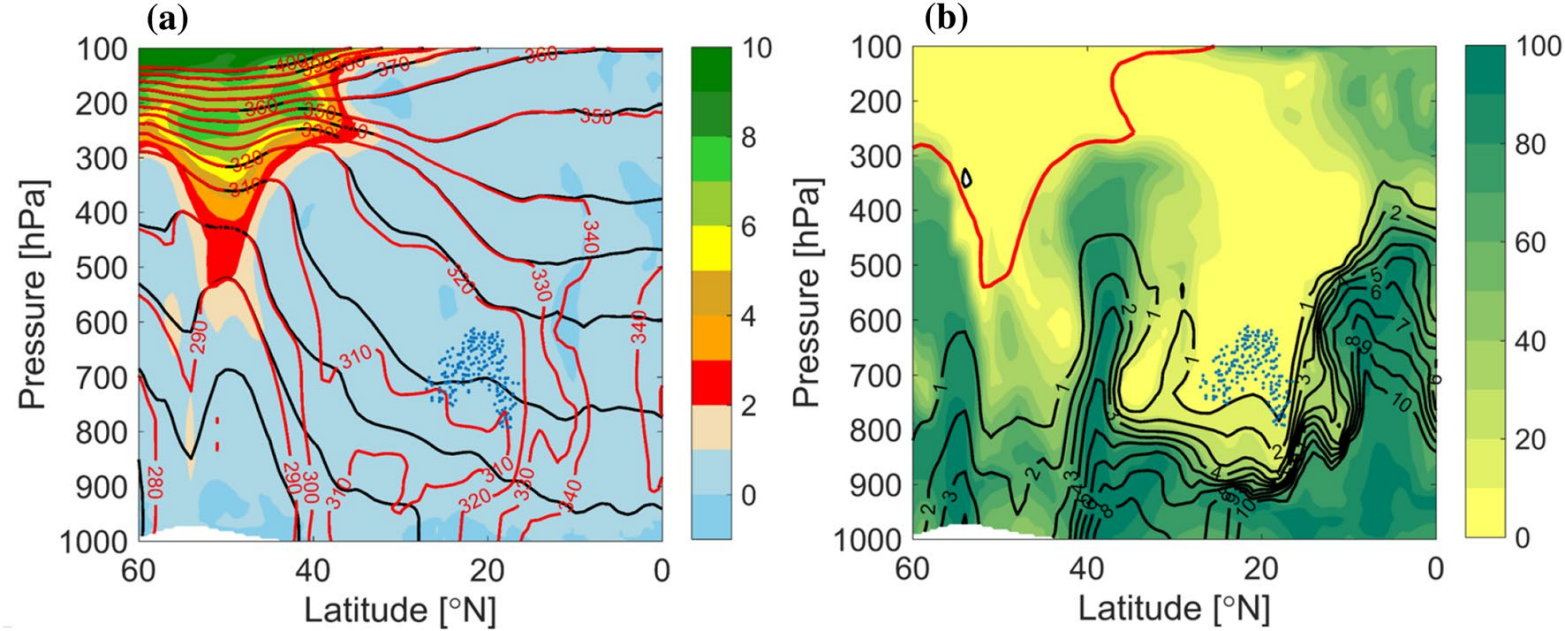
Vertical cross sections along the dashed line shown in Fig. 8. **a** PV (PVU, shaded), potential temperature (black contour) and equivalent potential temperature (K, red contour). **b** Relative humidity (%, shaded), specific humidity ($\hbox {g kg}^{-1}$, black contour) and 2-PVU contour (red). The blue points in both panels mark the intersection points of the DI trajectories with the vertical cross section, i.e., at any phase during their 48-h time of descent, and starting at different times

**Fig. 10:**
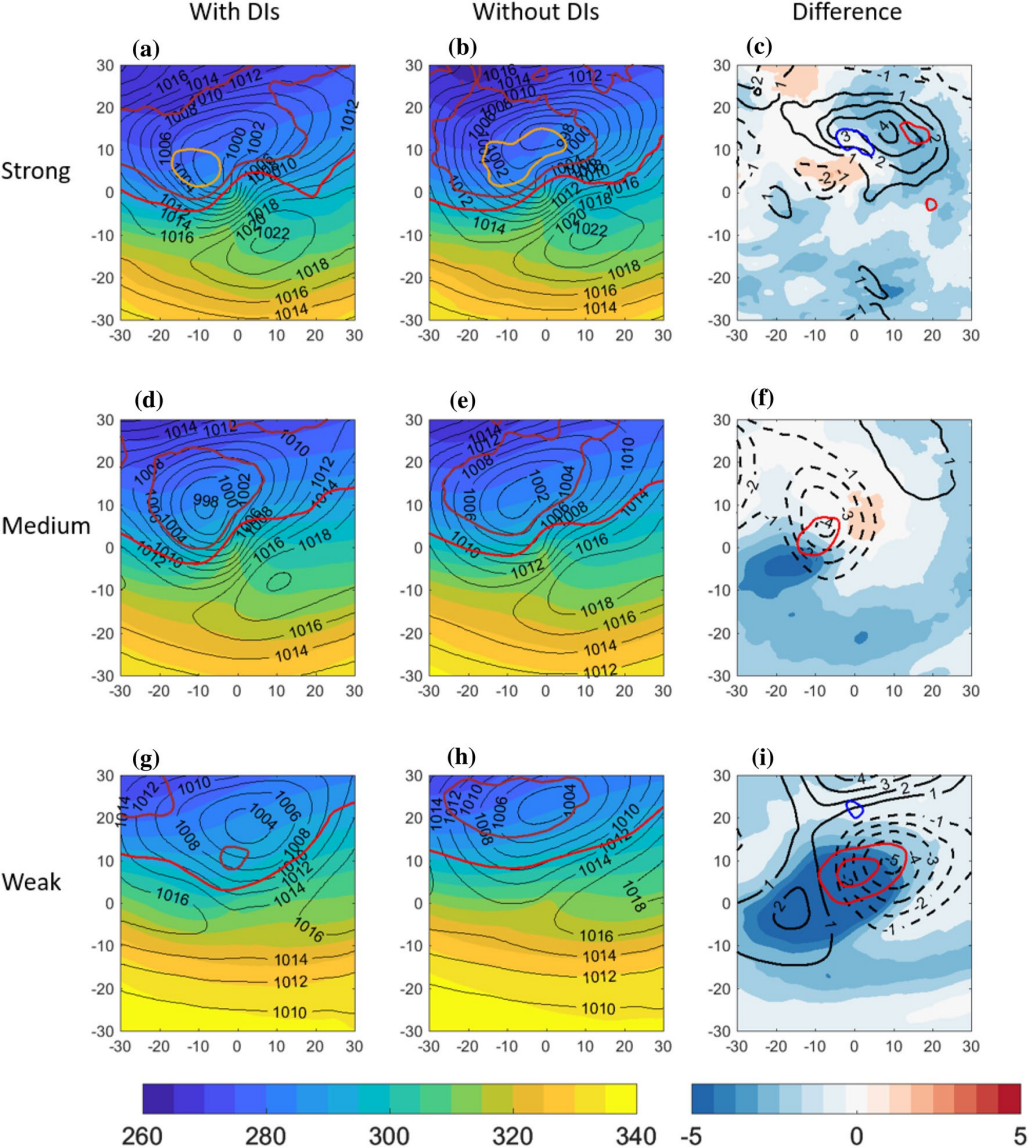
As Fig. 4, but for isolated North Pacific cold fronts

**Table 1:** Number of front objects within the Pacific study domain used for the composite analyses

Pacific Ocean	Strong front intensity	Medium front intensity	Weak front intensity
Trailing cold fronts	1184 (446)	4902 (2414)	691 (1436)
Isolated cold fronts	324 (174)	2678 (2427)	1846 (7930)

The numbers correspond to fronts that match with DIs, and the number in parentheses to fronts that do not match with DIs

**Table 2:** Number of front objects within the Atlantic study domain used for the composite analyses

Atlantic Ocean	Strong front intensity	Medium front intensity	Weak front intensity
Trailing cold fronts	1275 (781)	1802 (1442)	533 (1269)
Isolated cold fronts	276 (364)	716 (1143)	838 (6185)

The numbers correspond to fronts that match with DIs, and the number in parentheses to fronts that do not match with DIs

## Dry intrusions and trailing cold fronts

### An illustrative case: 3 January 2005

A prominent DI occurred during 1–5 January 2005, in which air descended slantwise from the upper troposphere in eastern Canada and reached the lower troposphere in the north western Atlantic Ocean, positioned behind a trailing front that obtained its maximum length during 3 January (Fig. 1). The front is connected to a deep cyclone to its north east, between southern Greenland and Iceland. The cyclone hosts a bent-back warm front as well, and an additional front feature within the cyclone area, regarded as a ‘central front’ in Catto and Raveh-Rubin (2019). Being by construction outside the cyclone area, the trailing front stretches further between two anticyclones to its east and west, on both sides of the Atlantic basin (Fig. 2a). The low-level DI object lies outside of the cyclone area, behind the front where its intensity is strongest, reaching 2.5 K/100 km (Fig. 2a). In the upper troposphere, the tropopause is lowered in a trough, below which a cold anomaly is located at low levels (Fig. 2b).

The relative location of the full DI (i.e., not only the low-level DI object) relative to the front at 00 UTC 3 January, is shown in Fig. 3 in a vertical cross section along the dashed line in Fig. 2. The location of the front is evident from the strong $\theta _{e}$ and $\theta$ gradients at $40^{\circ }\hbox {N}$, indicating a strongly baroclinic environment. The prominent upper-level trough reaches the 500-hPa level within the 40–45$^{\circ }\hbox {N}$ band. At this time, DI air spans from the area of the upper-tropospheric ridge at $55^{\circ }\hbox {N}$, below the trough and towards the front, at 900 hPa in the baroclinic zone. The moisture distribution shows the long vertical extent of the strong horizontal moisture gradients across the front, from 900 hPa to the upper troposphere ahead of the trough. The DI air is found in the driest regions, overriding the relatively moist boundary layer in the cold sector.

In the cold sector of the cyclone, intense surface fluxes into the atmosphere coincide with the DI object (Fig. 2c), consistent with the cyclone-relative location in Winschall et al. (2012), Aemisegger and Papritz (2018). Strong wind gusts occur in three main locations: in the cold conveyor belt jet (Hewson and Neu 2015), in the bent-back warm front region and in the northern portion of the trailing front, coinciding with intense precipitation there (Fig. 2c). Large-scale and convective precipitation are organized along the southwest-northeast oriented part of the front. The convective precipitation to the south of the front is seemingly related to the warm southerly advection (Fig. 2b, c).

To put this case study in a climatological perspective, the environment of similar trailing fronts with and without the presence of DIs behind them is examined in the next section.

### Trailing fronts composite

In order to generalize the characteristic environment of trailing fronts matching with DIs, compared to similar fronts without DIs, composite maps incorporate all trailing fronts in a confined geographical location in the central North Atlantic (Figs. 4, 5, 6) and Pacific (Supplementary Figures S1, S2, S3) Oceans. Since fronts occurring with DIs are significantly stronger (Catto and Raveh-Rubin 2019), here we control for the front intensity using separate composites for different intensities (see Sect. 2). By construction of the trailing front identification criterion and the compositing approach, a clear cyclonic field is indeed present with its centre around $-\,5^{\circ }\hbox {E}$ relative longitude and $5^{\circ }\hbox {N}$ relative latitude. The mean field for the 3 different front strength categories indicates that the stronger the front intensity, the larger and deeper the cyclone is in terms of central SLP, and the stronger the pressure gradients near [$0^{\circ }\hbox {E},0^{\circ }\hbox {N}$]. The front strength is directly visible as the area of maximal gradients of the $\theta _{e}$ field, indicating the mean position of the trailing fronts to the south and southwest of the cyclone. The equivalent potential temperature field also indicates that weak fronts occur on average in warmer regions at lower latitudes, consistent with the global climatological results in Catto and Raveh-Rubin (2019). The accompanying upper-tropospheric trough, shifted west of the cyclone centre by 5–10$^{\circ }$, is more pronounced the stronger the front. Considering the co-occurrence of DIs and trailing fronts, it is evident that fronts that match with DIs are associated climatologically with deeper cyclones, for a given front intensity. In these cases, the upper-level trough is enhanced as well (Fig. 4c, f, i). Although the difference in cyclone intensity is less pronounced in the weak fronts set, the anticyclone to its north east is deepened by more than 5 hPa on average when a DI is present (Fig. 4i), compared to weaker anticyclone differences for stronger fronts (Fig. 4c, f). Another anticyclone south west of the cyclone is enhanced in the presence of DIs. The overall stronger dipole structure of the SLP field in the vicinity of the front indicates stronger pressure gradient forces and stronger low-level northwesterly winds towards the front, in the presence of DIs. This finding is consistent with Tilinina et al. (2018), who recently highlighted the importance of the North American high located southwest of the turbulent heat fluxes maxima in the Western North Atlantic, in addition to a cyclone to their north east. In contrast, when DIs occur with fronts over the Pacific (Fig. S1), the northeastern anticyclone is enhanced mainly for the strongest fronts, while the south western anticyclone is enhanced in particular for weak fronts.

Since strong surface wind gusts and heat fluxes into the atmosphere are expected to be influenced by the presence of DIs, here we examine their composites for the three trailing front intensities (Fig. 5). First, fronts without DIs show monotonic increase of 10-m wind gusts with front intensity (Fig. 5b, e, h), peaking near the northeastern part of the front, at the composite centre, and to its west, i.e., where the cold conveyor belt is typically located (Hewson and Neu 2015; Raveh-Rubin and Wernli 2016). In addition, near strong and medium fronts, strong wind gusts show a secondary maximum over relative coordinates $10^{\circ }\hbox {E}, 10-15^{\circ }\hbox {N}$ (Fig. 5b, e). This secondary maximum is less pronounced for the Pacific front sets (Fig. S2). Sensible heat flux from the ocean to the atmosphere is expected to peak in the cold sector of the cyclone, as observed clearly in all the composite sets. A monotonic increase of sensible heat flux with front intensity is evident, possibly related to the colder air in the cold sector (Fig. 4). The composite latent heat flux does not follow linearly the front intensity. Considering the mean environmental conditions in the presence of DIs, a mean 10-m gusts increase of up to 3 m/s is evident west of the composite centre, roughly co-located with the increase in the magnitude of sensible heat flux into the atmosphere. Although these fields vary with front intensity on average, the additional DI occurs with the same mean increase in wind gusts independent of the front intensity. Rather differently, latent heat flux near weak fronts has a larger relative difference with the presence of DIs (of more than $60\hbox { W m}^{-2}$), compared to the climatological addition in the presence of DIs near strong fronts (reaching $40\hbox { W m}^{-2}$), possibly because of the warmer surface temperature near weak fronts.

The occurrence of precipitation in the frontal environment is expected to generally increase with front intensity (Catto and Pfahl 2013; Catto et al. 2015). In fact, the correlation between front intensity and precipitation was found to increase in the presence of DIs, in some regions more than two-fold, compared to fronts occurring without DIs (Catto and Raveh-Rubin 2019). It is still unclear to what degree the precipitation increase in the presence of DIs is a mere result of increased front intensity in such cases. To delineate these relationships, and learn about the spatial distribution of precipitation, here we examine composites of total 6-h precipitation and convective precipitation for the different front sets (Figs. 6, S3). Indeed, weaker precipitation is observed around the weak fronts, compared to stronger fronts in both ocean basins. Yet, no robust differences in mean precipitation amounts are seen between medium and strong front intensities. However, when occurring together with DIs, precipitation is enhanced for all front categories, with the most pronounced differences for medium front intensities. Interesting is that the convective fraction of the precipitation enhancement in the presence of DIs is higher for weaker fronts compared to medium, and further compared to strong fronts. Furthermore, the precipitation enhancement with DIs occurs to the north of weak trailing fronts, whereas for stronger fronts it has clear hook shape. South of the fronts, possibly on their warm side, a reduction of precipitation occurs in the presence of DIs, most notably south of weak fronts. This may be a result of the local subsidence conditions within the enhanced anticyclone.

## Dry intrusions and isolated cold fronts

### An illustrative case: 12 February 2005

A DI occurred north of an isolated front in the central north Pacific on 12 February 2005. The DI air descended while turning anticyclonically towards the subtropical latitudes, where an isolated front was identified at $15^{\circ }\hbox {N}$ (Fig. 7) with maximum intensity of 1.3 K/100km. The front is isolated from a trailing front to its north (which matches with another, small DI object) and another trailing front to its northeast (which occurs without a DI), and is clearly disconnected from the deep midlatitude cyclone in the northwest Pacific Ocean (Fig. 8). The horizontal and vertical distributions of potential temperature and the humidity fields (Figs. 8b, 9) indicate that the strong gradients of $\theta _{e}$ stem from the large moisture contrast between the subtropical and tropical airmasses between 15 and 20$^{\circ }\hbox {N}$, in contrast to the clear baroclinic environment to its north at $40^{\circ }\hbox {N}$ (Fig. 9a), similar to the ‘dry line’ described in Arnup and Reeder (2007) for Northern Australia. The DI air is located behind the front, away from the upper-tropospheric trough and the baroclinic zone at higher latitude.

The front impact in terms of surface fluxes, 10-m wind gusts and precipitation are rather mild, with wind gusts below 15 m/s, and precipitation slightly exceeding 5 mm/6h, in comparison to higher impact along the neighboring trailing fronts (Fig. 8c). Thus, the isolated front is located on the periphery of a trailing front of weak-medium intensity, which is in turn, associated with heavy precipitation.

### Isolated fronts composite

Generalized understanding of the environment of isolated fronts is given by compositing all isolated front objects in the central Pacific and Atlantic Oceans, for different sets of front intensities, with and without the co-occurrence of DIs in their close vicinity. The composite fields demonstrate clear, dynamically meaningful patterns. The composite SLP for all front sets indicates that although, by definition, the isolated fronts are clearly disconnected from cyclones, there is a clear signature of low mean SLP north of the front northeastern corner (Figs. 10, S4). Notably though, the low-pressure systems are located further to the north, especially in the Atlantic (Fig. S4), and span a wider zonal extent, compared to the parent cyclone of trailing fronts (Fig. 4), suggesting that they reflect the North Pacific/Atlantic climatological maximum (Fig. 2 in Catto and Raveh-Rubin 2019). Considering first the variability with respect to front intensities, very different low- and upper level flow configurations are evident. Stronger isolated fronts are on average closer to the low-pressure systems to their north, while weak fronts are found further south, at warmer $\theta _{e}$ regions. The southern location of the weak fronts is also evident from the larger distance from the stratospheric high potential-vorticity (PV) reservoir, compared to stronger fronts. Contrasting these features with and without the presence of DIs, additional changes arise, differently for each intensity category. Strong fronts with DIs occur near a weaker cyclone, but closer to it, accompanied by increased upper-tropospheric jet waviness, compared to strong fronts without DIs (Fig. 10c). Fronts of medium intensity, however, occur nearer to a cyclone, which is on average also stronger, in the presence of DIs compared to cases where DIs are absent (Fig. 10f). The difference plot (Fig. 10f) also suggests enhanced baroclinic growth with DIs, with a positive upper-level PV anomaly and low-level cold and warm advection behind and ahead of the cyclone, respectively. Pronounced differences occur when DIs match with weak isolated fronts (Fig. 10i). With DIs, there is a prominent signature of an upper-level trough, compared to almost zonal conditions when DIs are absent. At the lower troposphere, lower SLP is found to the east of the trough and higher SLP to its southwest, resulting in stronger pressure gradient force and large cold and dry advection, exceeding − 9 K difference of $\theta _{e}$ on the 850-hPa surface.

**Fig. 11:**
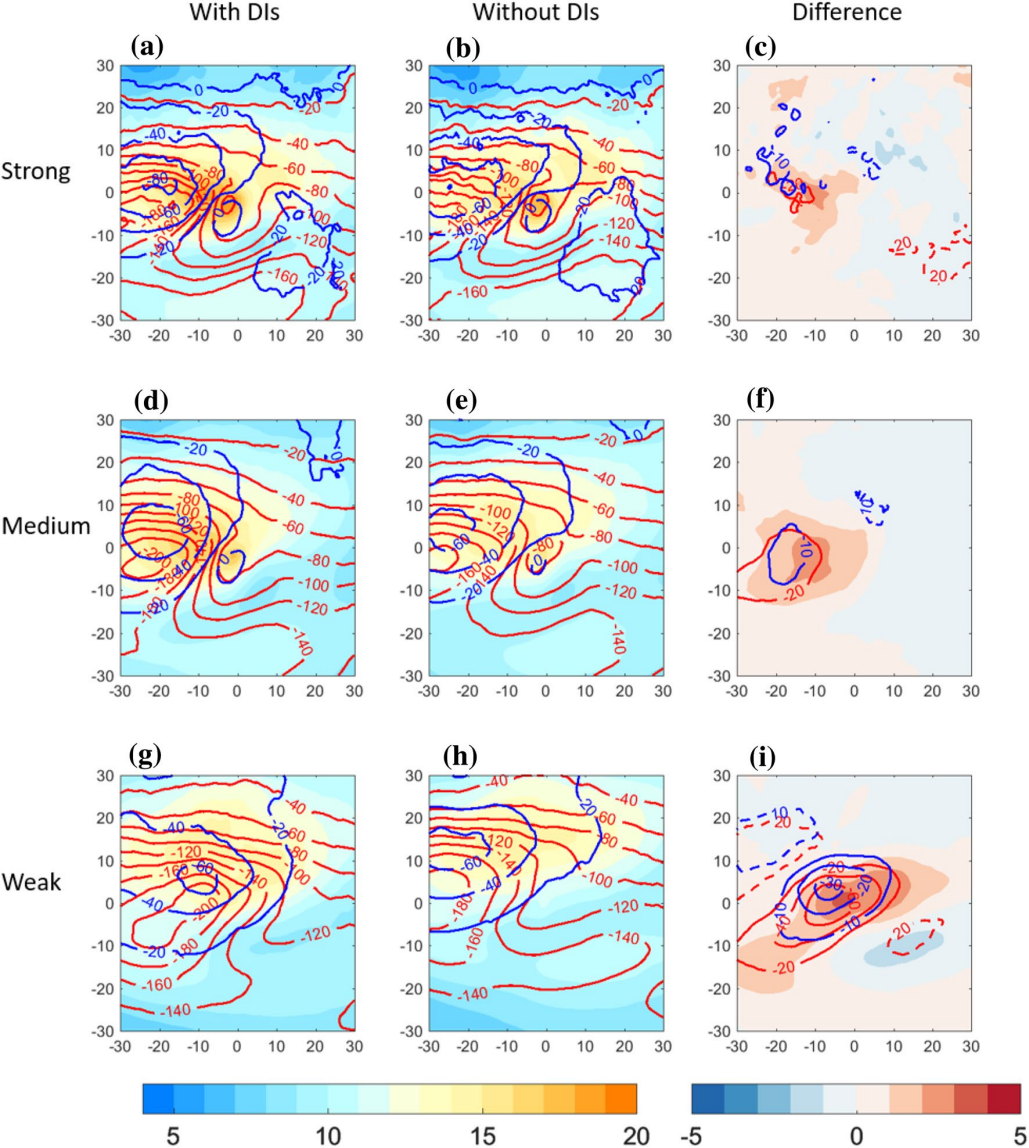
As Fig. 5, but for isolated North Pacific cold fronts

Wind gusts at 10 m near isolated fronts are highest on average at and to the north and west of the strongest fronts, extending further to the north and north east of the weak fronts (Figs. 11, S5), consistent with the location of the composite SLP minima. The additional presence of DIs is associated with enhancement of the gusts coherently for all front intensities, by 2–4 m/s. Surface fluxes generally peak to the west of the fronts, with sensible heat flux into the atmosphere being higher for stronger fronts. Latent heat fluxes do not change monotonically with front intensity, but in the presence of DIs, both sensible and latent heat fluxes increase, especially for weak fronts, around which the magnitude of sensible and latent heat flux increase on average by 10–30 $\hbox { W m}^{-2}$ and 20–60 $\hbox { W m}^{-2}$, respectively (Fig. 11i).

**Fig. 12:**
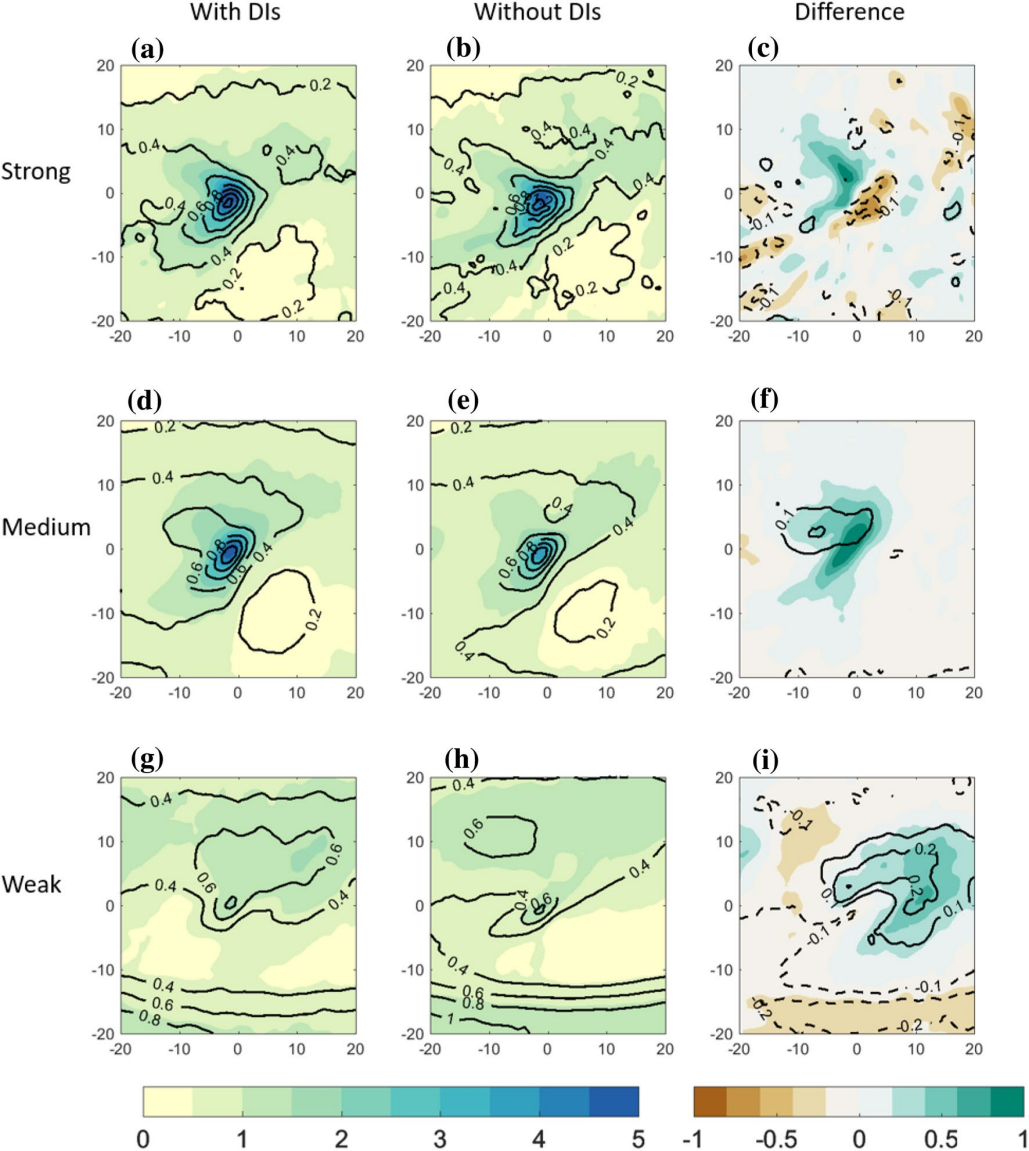
As Fig. 6, but for isolated North Pacific cold fronts

Precipitation near isolated fronts peaks in a more confined region around the front, compared to trailing fronts (Figs. 6,12, and S6, S3). Both total precipitation and convective precipitation are tightly linked with the front intensity. With DIs, there are climatologically increased precipitation amounts, most notable for N. Pacific fronts of medium intensity. However, near strong fronts the dipole structure in Fig. 12c suggests that non-convective precipitation is enhanced on the NW side of the front, and (convective) precipitation is suppressed on the SE side. Weak isolated fronts are climatologically associated with light and localized convective precipitation (Fig. 12g, h). Yet, coherent shifts of the distribution of precipitation away from the front are evident in the presence of DIs (Fig. 12i), suggestive of other large-scale factors in play. The precipitation increase northeast of the weak fronts is concurrent with increased surface fluxes there (Fig. 11i, note the different spatial extent of the composites) as well as decreased static stability below the lowered tropopause there (Fig. 10i). The zonal band of suppressed precipitation in the south occurs in an area of diffluent flow with a cold anomaly at the 850-hPa level, thus inducing an anomalously stable condition, compared to similar fronts that occur without the DIs and the aforementioned upper-level forcing.

Overall, isolated fronts entail diverse environmental changes when occurring together with DIs, depending on the front strength. Strong fronts with DIs are associated with a *weaker* cyclone in its vicinity, as well as a shift of precipitation northwestwards and a decrease of convective precipitation, compared to similar fronts without DIs (the latter occur most rarely, see Table 1). Isolated fronts of medium intensity are characterized by environmental characteristics comparable to those of trailing fronts (Sect. 3). However, weak isolated fronts occur further equatorward. Although they occur most commonly without DIs, in the cases when DIs are present, notable differences emerge for all three front intensity categories. Namely, enhanced interaction with the midlatitude upper-troposphere, inducing strong cold and dry low-level advection, enhanced surface heat and moisture fluxes as well as more intense wind gusts.

## Summary and discussion

**Fig. 13:**
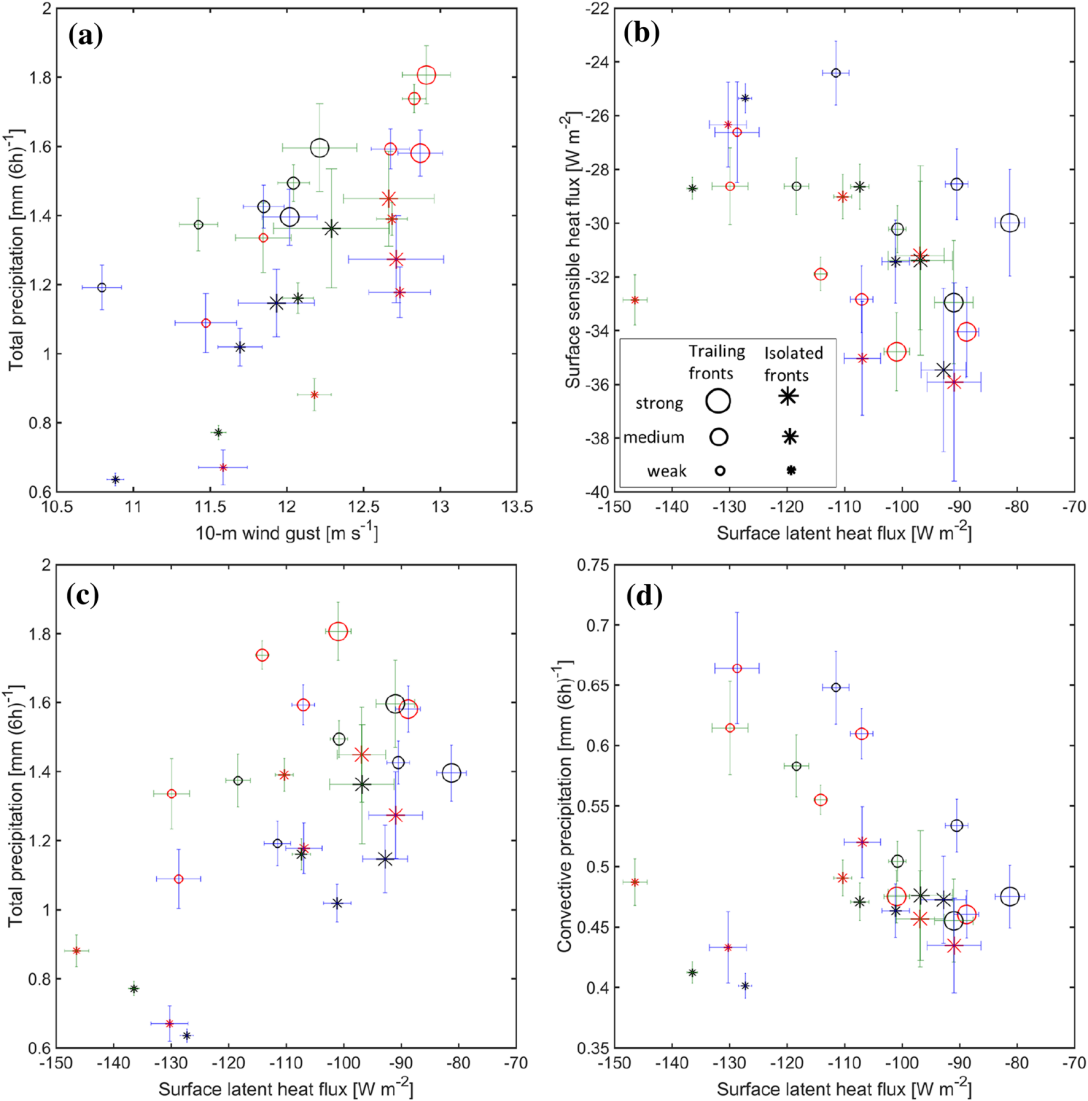
Summary statistics of the composite analysis for both N. Atlantic and N. Pacific fronts. Composite mean and error bars showing the standard error of the mean of different variables are plotted for different composited front sets, and marked in blue for N. Atlantic, and green for N. Pacific front sets, with DIs (red) and without DIs (black). Trailing fronts are shown in circles, isolated fronts are shown in asterisks, and the marker size indicates the front intensity, as shown in the legend in panel (**b**). **a** 10-m wind gusts [$\hbox {m s}^{-1}$] and total precipitation [$\hbox {mm (6h)}^{-1}$]; **b** surface sensible and latent heat fluxes [$\hbox {W m}^{-2}$]; **c** surface latent heat flux [$\hbox {W m}^{-2}$] and total precipitation [$\hbox {mm (6h)}^{-1}$]; and **d** surface latent heat flux [$\hbox {W m}^{-2}$] and convective precipitation [$\hbox {mm}\, \hbox {(6h)}^{-1}$]. The composite mean and standard error of the mean are first calculated locally at each grid point, based on all composite members, and further spatially averaged over the domain of the centralized composites: $\pm \, 10^{\circ }$ relative latitude and longitude for total and convective precipitation; $\pm \, 20^{\circ }$ relative latitude and $-30^{\circ }$ to $+\,10^{\circ }$ relative longitude for surface fluxes and 10-m wind gust

In this study fronts trailing from cyclones are distinguished from fronts that are isolated from cyclones, allowing a novel targeted examination of the the characteristic dynamical and thermodynamical environment of each type of front with (and without) DIs. Separate composite analysis of N. Atlantic and N. Pacific fronts suggests robust characteristics with minor variation across ocean basins. Insight on the environment of fronts and their impact, as well as how these are shaped in the presence of DIs can be gained from combining all front sets together (Fig. 13). The standard error of the composite mean indicates that there is larger variability the stronger the fronts. The largest standard error of the composite mean occurs for the strong isolated fronts, which constitute the smallest number of individual fronts (Table 1). The occurrence of such fronts with and without DIs does not exhibit significantly different mean surface fluxes and precipitation. On the other hand, weak isolated fronts compose a large set of fronts, where, importantly, the associated dynamical and thermodynamical environment significantly differ in the presence of DIs.

For a given front intensity, precipitation is stronger near trailing fronts, compared to isolated fronts, a difference that is most marked for weak fronts (Fig. 13a). Mean precipitation and 10-m wind gusts in the vicinity of fronts both increase with front intensity, consistent with Catto and Pfahl (2013). This is valid for the composite means of both trailing and isolated fronts, and when considering separately fronts with and without matching DIs. Moreover, mean precipitation and gusts are more intense when fronts are accompanied by DIs compared to similar fronts that occur without DIs (Fig. 13a), with an exception of weak trailing fronts. A second exception to this general tendency is isolated strong and medium fronts which have comparable mean precipitation and gusts in the presence of DIs.

DIs occur behind the cold fronts, i.e., on their cold and dry side, where surface fluxes into the atmosphere are maximal, while the mixing of DI air into the PBL presumably affects the intensity of both fluxes. Indeed, both distributions of sensible and latent heat fluxes behind fronts generally enhance (are more negative) in the presence of DIs (Fig. 13b). Anti-correlation emerges between mean sensible and latent heat fluxes. Enhanced ocean evaporation is expected to contribute to the availability of moisture for precipitation (with certain spatio-temporal biases due to horizontal advection). Indeed, although their maxima locations are generally shifted by $\sim$ 10$^{\circ }$–20$^{\circ }$, this correlation holds when comparing the composite means of the surface latent heat flux and convective precipitation (Fig. 13d), with an exception of weak isolated fronts that have the lowest convective precipitation but strongest latent heat flux (note that they occur at lower latitudes). Here too, the occurrence of fronts with DIs is accompanied by stronger latent heat fluxes and stronger convective precipitation for all front sets, including the weak isolated fronts (but excluding the strongest isolated fronts, which have the highest variability and comparable fluxes and precipitation means with and without DIs).

Although when occurring with DIs most front sets are accompanied by stronger latent heat fluxes and enhanced mean total precipitation compared to similar fronts without DIs, a rather surprising relationship yet emerges: a negative correlation generally exists between ocean moisture fluxes and total precipitation among all front sets (Fig. 13c). As such, although weaker fronts are associated with enhanced surface evaporation, mean total precipitation is lower in their vicinity, as already noted in Sect. 4.2. Several explanations for this behavior may include: (1) The composite analysis here suggests that weaker fronts are associated with weaker cyclones (in terms of mean central SLP), even for isolated fronts which are far and disconnected from the cyclone area. Consistently, there is shallower upper-tropospheric support for baroclinic growth, compared with stronger fronts. Both trailing and isolated weak fronts occur with reduced pressure gradients and associated warm and moist advection, compared with stronger fronts, necessary for the moisture flux convergence and uplift. (2) Contrary to the rather local coupling between moisture fluxes and convective precipitation, additional contribution from advection are involved in the generation of the total precipitation. Near cyclones, these include predominant slantwise ascent in the warm conveyor belt which in principle leads to non-local spatio-temporal relationship between moisture uptake and precipitation (Pfahl et al. 2014). (3) Weaker fronts are preferentially located at lower latitudes, where latent heat fluxes are higher, and precipitation is not limited by moisture availability (Pfahl and Sprenger 2016). (4) Strong heat fluxes in the cold sector act to weaken the temperature gradients across the cold front, resulting in the monotonic reduction of latent heat fluxes with increasing front intensity. The latter point is consistent with the numerical case study in Gozzo and Da Rocha (2013) and with the increased asymmetry of cloudiness between the warm and cold cyclone sectors in cases of enhanced convection (Naud et al. 2015).

It is important to note that it is often misleading to deduce causal or even correlative relationships among composite mean values, as these are influenced by additional environmental factors and may not infer an actual correlation among the individual events within each set of fronts. An additional caveat of the statistical climatological study (Catto and Raveh-Rubin 2019) and the current feature-based composite analysis is the lack of distinction between different stages of the front life cycle. For example, the diurnal cycle was shown to affect subtropical fronts (Smith et al. 1995; Reeder et al. 2000; Thomsen et al. 2009), while the air–sea interaction and precipitation strongly depend on the baroclinic life cycle (Kuo et al. 1991; Catto 2016). Tracking of the fronts may provide further insight on the timing of DI-front co-occurrence during the front life cycle, such as the possible evolution of broken trailing fronts segments into isolated front features, or the timing of the front match with the DIs through their mutual life cycles.

The current study provides insight into the environment of the co-occurrence of DIs and cold fronts in the Northern Hemisphere, both in the context of extratropical cyclones (Browning 1997) and as a feature separate from the baroclinic environment. In terms of composite means, the environmental changes with and without the presence of DIs are comparable in magnitude and at times larger that the differences between different front types. This stresses the central role that DIs play in the variability of wind gusts, precipitation, surface fluxes and low-level $\theta _{e}$ distribution in the vicinity of the fronts. In fact, some environmental variables, namely surface heat fluxes, heat and moisture transport across the front, flow deformation and along-front transport, were recently shown to explain the majority of front variability (Spensberger and Sprenger 2018), the first three of which are potentially related to DIs.

The composite results are consistent with Catto and Pfahl (2013) who showed stronger front intensities for extreme precipitation. However, Pfahl and Wernli (2012) showed that cyclones are not deeper than usual during extreme precipitation events. Consistently, the composite analysis here suggests that although cyclone intensity correlates with their trailing front intensity and surrounding precipitation, the differences between the medium and the strong front intensity categories are very small, and partly lie within the range of composite mean error. Building on the current study, in-depth investigation into the climatological relationship between cyclone and front characteristics, their impact and latitudinal dependence is currently underway.

## Electronic supplementary material

Below is the link to the electronic supplementary material.

Supplementary material 1 (pdf 2682 KB)

Supplementary material 1 (gif 6137 KB)
