# Supplementary material for: Climatology and dynamics of the link between dry intrusions and cold fronts during winter, Part II: Front-centred perspective
Source: Clim Dyn. 2019 May 7;53(3):1893–909. doi: 10.1007/s00382-019-04793-2 (PMC6647394; doi:10.1007/s00382-019-04793-2)
Supplement: Supplementary file 1 — Supplementary material 1 (pdf 2682 KB) [file 382_2019_4793_MOESM1_ESM.pdf]

Climatology and Dynamics of the Link Between Dry Intrusions and Cold Fronts During Winter, Part II: Front-Centered Perspective

Climate Dynamics

Shira Raveh-Rubin, Jennifer L. Catto

Author affiliation:

Shira Raveh-Rubin: Department of Earth and Planetary Sciences, Weizmann Institute of Science, Rehovot, Israel; email: shira.raveh-rubin@weizmann.ac.il.

Jennifer L. Catto: College of Engineering, Mathematics and Physical Sciences, University of Exeter, UK

Supplementary animation

The animation shows the identified features for each 6-h step during January 2005. The gridded cold fronts are shown by the yellow/red grid boxes, with the darker colors indicating stronger wet bulb potential temperature gradients. Trailing front masks are enclosed within blue contours, isolated fronts in magenta, and central fronts in red. Green masks indicate low-level DI objects, and the cyclone masks used for determining the front type are shown in grey. Bold contours indicate matched objects.

Supplementary figures

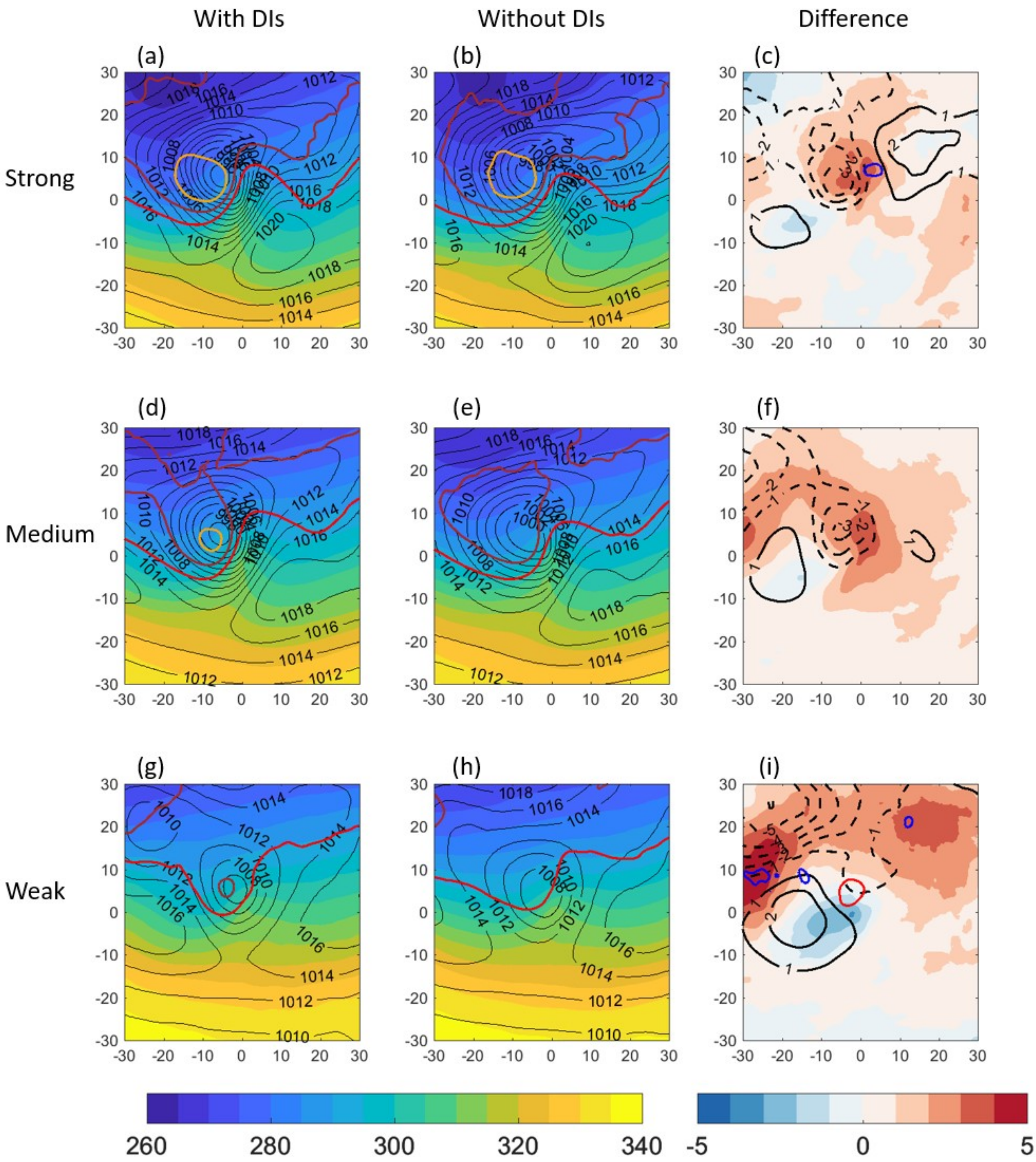

Figure S1. Centered composite of North Pacific cold trailing fronts, centered around their NE corner at [0°E,0°N] relative longitude and latitude, for different front sets. Top row: strong front intensity; Middle row: medium front intensity; Bottom row: weak front intensity. Left column: composite of cold trailing fronts that match with a DI; Middle column: cold trailing fronts that do not match with a DI. Right column: difference between composite means [with DIs]-[without DIs]. Plotted for the full fields are θ_e on 850 hPa (K, shaded), sea-level pressure (hPa, black contour), and potential vorticity on 300 hPa (2 PVU in red, 3 PVU in brown and 4 PVU in orange). Plotted for the difference fields are θ_e on 850 hPa (K, shaded), SLP (hPa, solid and dashed black lines, zero line omitted), and PV on 300 hPa (PVU, red contours for positive values above 0.5 PVU at 0.5 PVU intervals, and blue contours for the negative counterpart, 0-contour omitted).

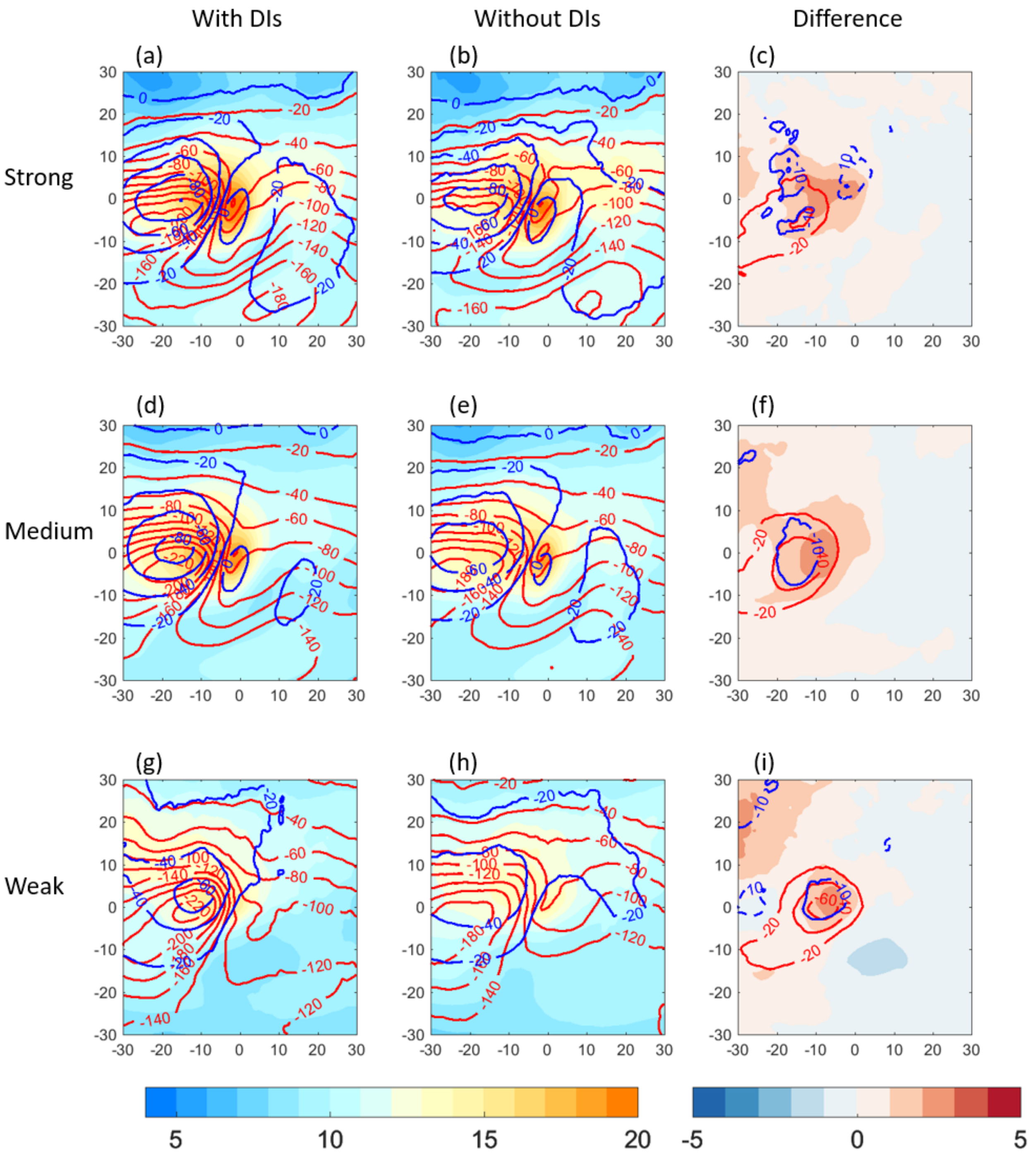

Figure S2. Centered composite of North Pacific cold trailing fronts, centered around their NE corner at [0°E,0°N] relative longitude and latitude, for different front sets. Top row: strong front intensity; Middle row: medium front intensity; Bottom row: weak front intensity. Left column: composite of cold trailing fronts that match with a DI; Middle column: cold trailing fronts that do not match with a DI. Right column: difference between composite means [with DIs]-[without DIs]. Plotted for the full fields are maximum 10-m wind gust (m s^{-1}, shaded), surface sensible heat flux (W m^{-2}, blue) and surface latent heat flux (W m^{-2}, red). Plotted for the difference fields are maximum 10-m wind gust (m s^{-1}, shaded), surface sensible heat flux (W m^{-2}, blue contour with 10 W m^{-2} interval, solid line for negative and dashed line for positive values) and surface latent heat flux (W m^{-2}, red contour with 20 W m^{-2} interval, solid line for negative and dashed line for positive values).

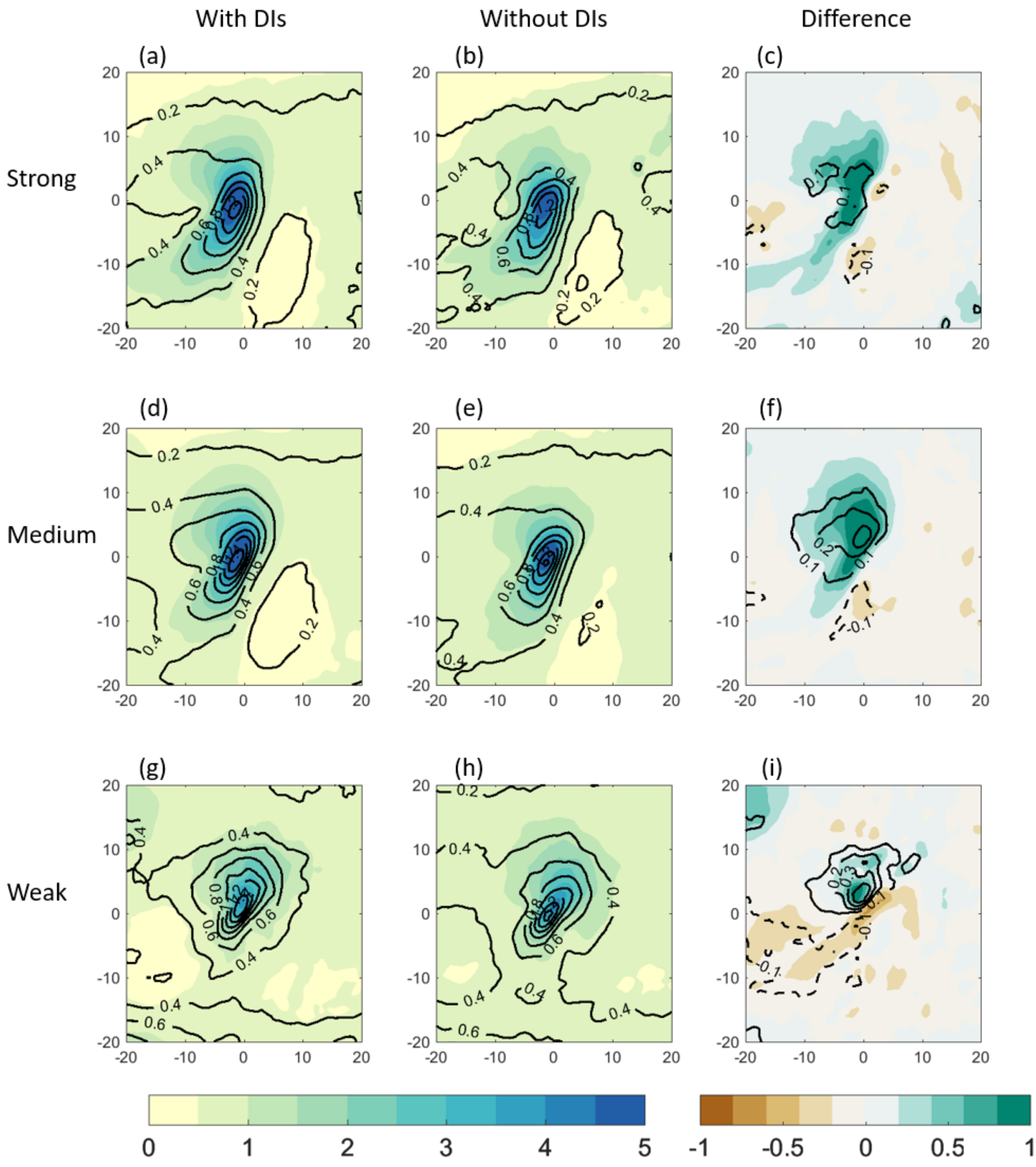

Figure S3. Centered composite of North Pacific cold trailing fronts, centered around their NE corner at [0°E,0°N] relative longitude and latitude, for different front sets. Top row: strong front intensity; Middle row: medium front intensity; Bottom row: weak front intensity. Left column: composite of cold trailing fronts that match with a DI; Middle column: cold trailing fronts that do not match with a DI. Right column: difference between composite means [with DIs]-[without DIs]. Plotted are total precipitation (mm $(6h)^{-1}$, shaded) and convective precipitation (mm $(6h)^{-1}$in black).

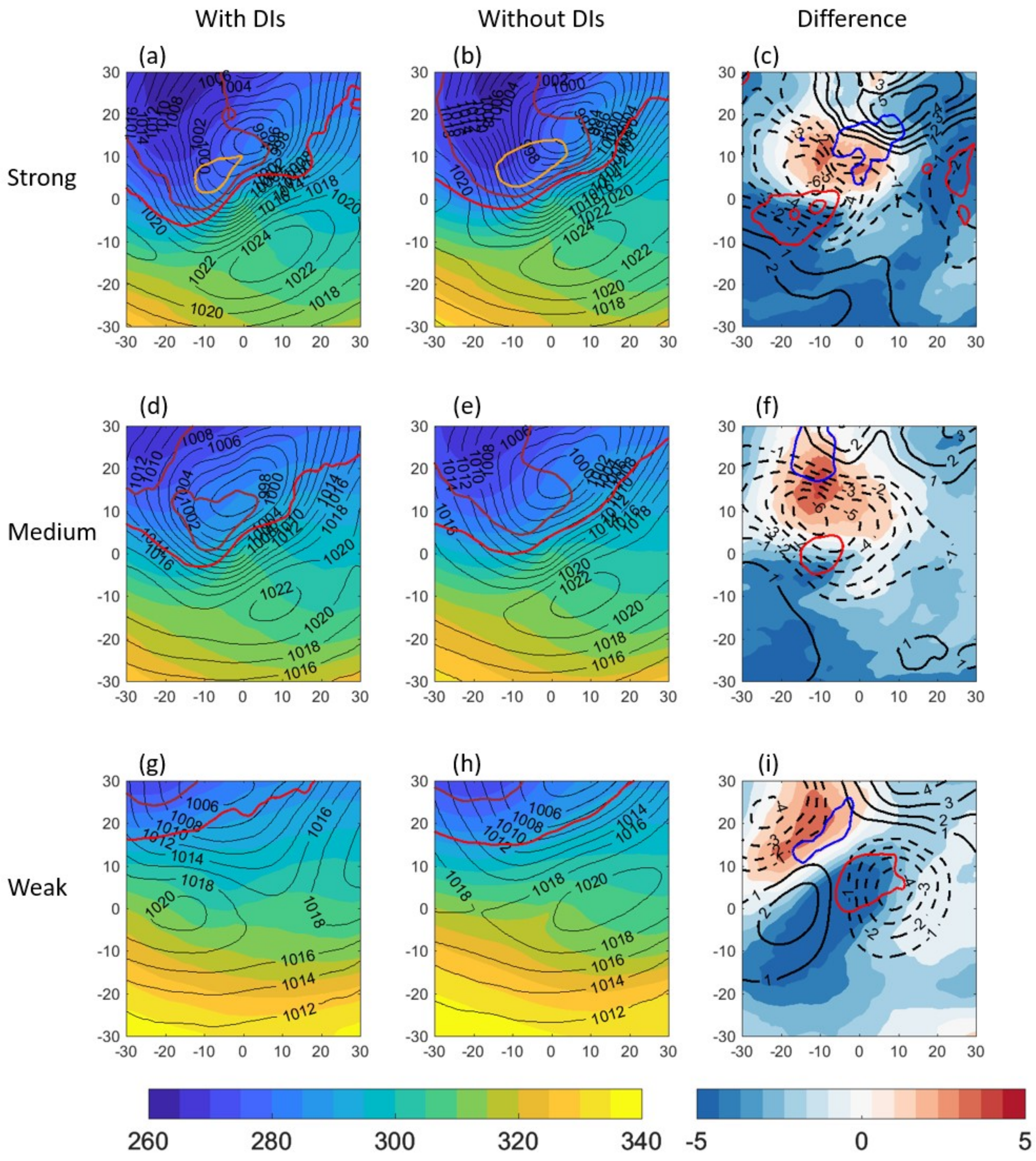

Figure S4. As Fig. S1, but for isolated Atlantic cold fronts.

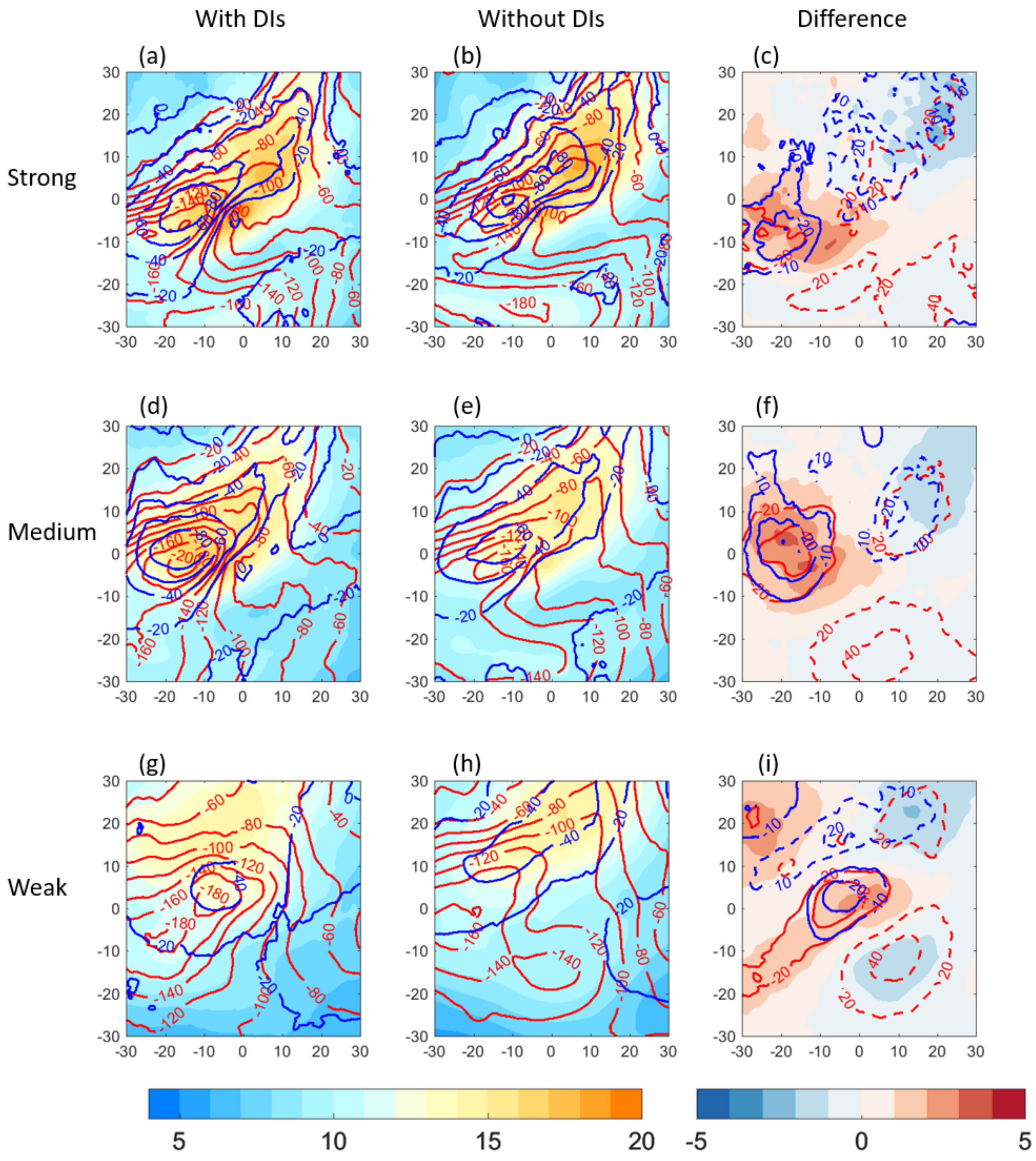

Figure S5. As Fig. S2, but for isolated Atlantic cold fronts.

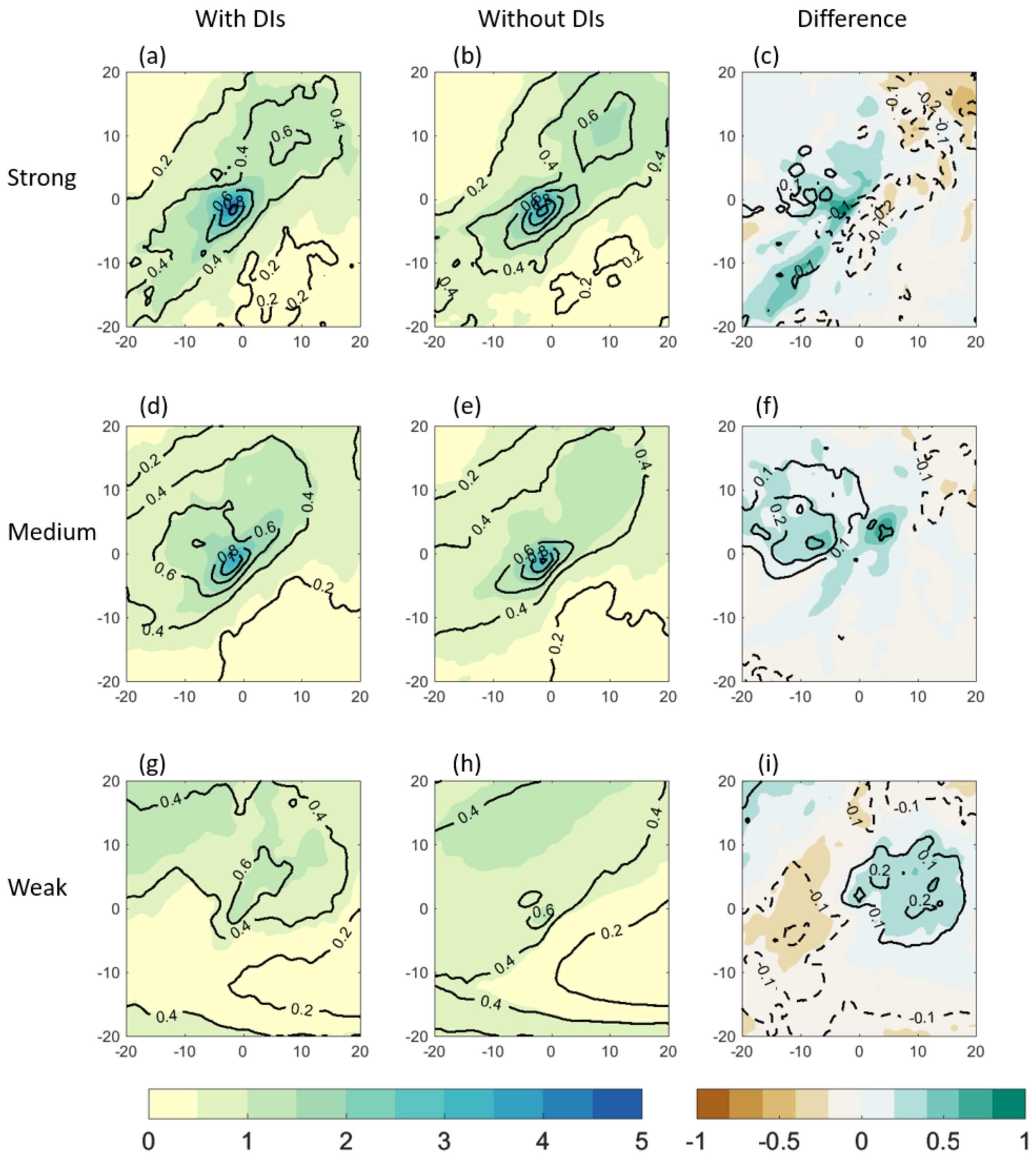

Figure S6. As Fig. S3, but for isolated Atlantic cold fronts.
