# Supplementary figures and images for: Climatology and dynamics of the link between dry intrusions and cold fronts during winter, Part II: Front-centred perspective
Source: Clim Dyn. 2019 May 7;53(3):1893–909. doi: 10.1007/s00382-019-04793-2 (PMC6647394; doi:10.1007/s00382-019-04793-2)

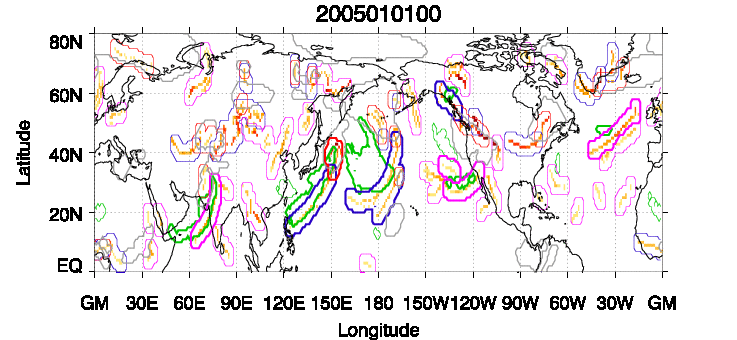

Supplement: Supplementary file 2 — Supplementary material 1 (gif 6137 KB) [file 382_2019_4793_MOESM2_ESM.gif]
